# Atlas of Ohio Aquatic Insects: Volume II, Plecoptera

**DOI:** 10.3897/BDJ.4.e10723

**Published:** 2016-11-16

**Authors:** R. Edward DeWalt, Scott A. Grubbs, Brian J. Armitage, Richard W. Baumann, Shawn M. Clark, Michael J. Bolton

**Affiliations:** ‡ University of Illinois, Champaign, United States of America; § Western Kentucky University, Department of Biology and Center for Biodiversity Studies, Bowling Green, Kentucky, United States of America; | Instituto Conmemorativo Gorgas de Estudio de la Salud, Ciudad de Panamá, Panama; ¶ Monte L. Bean Life Science Museum, Brigham Young University, Provo, Utah, United States of America; # Ohio Environmental Protection Agency, Division of Surface Water, Groveport, Ohio, United States of America

**Keywords:** Ohio, U.S.A., Plecoptera, stoneflies, museum data, distribution, emergence, stream size

## Abstract

**Background:**

We provide volume II of a distributional atlas of aquatic insects for the eastern USA state of Ohio. This treatment of stoneflies (Plecoptera) is companion to Armitage et al. (2011) on caddisflies (Trichoptera). We build on a recent analysis of Ohio stonefly diversity patterns based on large drainages (DeWalt et al. 2012), but add 3717 new records to the data set. We base most analyses on the United States Geological Survey Hierarchical Unit Code eight (HUC8) drainage scale. In addition to distributional maps for each species, we provide analyses of species richness versus HUC8 drainage area and the number of unique locations in a HUC8 drainage, species richness versus Ohio counties, analyze adult presence phenology throughout the year, and demonstrate stream size range affiliation for each species.

**New information:**

This work is based on a total of 7797 specimen records gathered from 21 regional museums, agency data, personal collections, and from the literature Table 1. To our knowledge this is the largest stonefly data set available for a similarly sized geopolitical area anywhere in the world. These data are made available as a Darwin Core Archive supported by the Pensoft Integrated Publishing Toolkit (DeWalt et al. 2016b). All known published papers reporting stoneflies from Ohio are detailed in Suppl. material 1. We recovered 102 species from Ohio, including all nine Nearctic families Table 2​. Two species were removed from the DeWalt et al. (2012) list and two new state records added. Perlidae (32 spp.) was most speciose, compared to the low diversity Pteronarcyidae (2 spp.) and Peltoperlidae (1 sp.). The richest HUC8 drainages occurred in northeastern, south-central, and southern regions of the state where drainages were heavily forested, had the highest slopes, and were contained within or adjacent to the unglaciated Allegheny and Appalachian Plateaus. Species poor drainages occurred mainly in the northwestern region where Wisconsinan aged lake plains climaxed to an expansive wooded wetland, the Black Swamp. The unglaciated Lower Scioto drainage (72 spp.) in south-central Ohio supported the greatest species richness. There was no relationship between species richness and HUC8 drainage size, but the number of unique locations in a drainage strongly related to species richness. All Ohio counties were represented in the data set with Hocking County (59 spp.) of the Lower Scioto drainage being the richest and most heavily sampled. Adult presence phenology was influenced by phylogenetic relationships such that the superfamily Nemouroidea (Capniidae, Leuctridae, Nemouridae, and Taeniopterygidae) generally emerged in winter and spring while the superfamilies Pteronarcyoidea (Pteronarcyidae, Peltoperlidae) and Perloidea (Chloroperlidae, Perlidae, Perlodidae) emerged later, some species continuing emergence through summer months. Species often occupied specific stream size ranges, while others were generalists. Two species once histrorically abundant in the western Lake Erie Bass Islands no longer reside there. Each of the 102 species is discussed in detail, including several that require additional collecting efforts to confirm their identities, presence, and distribution in Ohio.

## Introduction

Stoneflies (Insecta: Plecoptera) are one of many faunal groups that reflect the historical geography of Ohio. The presence and distribution of stoneflies in Ohio demonstrate not only the results of the terraforming effects of Quaternary glaciation, but also the various invasion routes available in preglacial epochs. For example, the preglacial (and pre-Ohio River) Teays River drainage, originated in western North Carolina and provided access to Ohio, Indiana and Illinois (Hansen 1995, King 1983, Ver Steeg 1946) Whereas, this extensive drainage is buried under 500 feet or more of glacial till from central Ohio westward, at least a few of the stoneflies which colonized Ohio using this route may have found refuge in the Western Allegheny and Appalachian Plateaus of eastern and southeastern Ohio during the glacial epochs. Others recolonized from refugia in the Cumberland Plateau, Southern Appalachian Mountains, and possibly the Ozark Mountains (Pessino et al. 2014, Ross et al. 1967). The series of glacial events flattened most of northwestern and western Ohio, down to the Cincinnati area, creating lake, till, and drift plains, bogs, and fens. In northwestern Ohio the Black Swamp (a.k.a. Great Black Swamp), a wooded wetland complex, was formed atop lake plains of ancient glacial Lake Maumee (Kaatz 1955). This area was not drained until the second half of the 19^th^ century. The sum of these historical events, in conjunction with more recent natural and human-caused factors, in large part, explains Ohio’s stonefly fauna today.

Properly maintained natural history (museum) collections provide a permanent record of life on Earth (Mehrhoff 1997). The use of information technology, coupled with data standards (unique identifiers, georeferencing, and data sharing formats), has recently improved access and manipulation of the information. The specimens and their labels place a species in space and time, making natural history collections useful not only for such typical purposes as systematics research, but also as a source of verifiable data to examine range changes over time, to study the effects of environmental degradation, and to predict the extent and severity of invasions of exotic species. We may also extract from these data life history information, habitat requirements, understand the imperilment of species at multiple scales, plan for restoration activities, and examine relationships of distributions to landscape and species trait constraints.

Given that stoneflies are one of the most sensitive indicators of change in habitat and water quality (Stewart and Stark 2002), they are important targets for digitization of museum specimen records and ecological analyses based on those records. Much work to this affect has already occurred in Illinois. Favret and DeWalt (2002) and DeWalt et al. (2005) amassed 5117 records for Illinois, demonstrating that 28% of the original fauna had been extirpated from the state, that every region of the state experienced losses, and that the data were sufficient to build state level conservation statuses for each species. Another direct result of compiling these large data sets was the Cao et al. (2013) predictions of pre-European settlement distribution and richness patterns of Illinois stoneflies at the USGS HUC12 watershed scale . Other studies in the USA that have benefited from accumulating stonefly museum data include DeWalt et al. (2012) for Ohio, DeWalt and Grubbs (2011) for Indiana, and Grubbs et al. (2013b) for Michigan. In Europe, Bojková et al. (2012) in the Czech Republic used 170 fixed sites to examine changes in the assemblage from the middle 20^th^ century. Additionally, RWB and colleagues are working on an atlas of stoneflies for Nevada, USA with a greatly expanded species list, distributional maps, specimen images, and a comprehensive database slated for publication in spring, 2017.

Prior to DeWalt et al. (2012), Ohio’s stonefly fauna had been studied in a piecemeal fashion. Walker (1947) provided a southeastern Ohio treatment, including a few records from the southwestern and northwestern corners of the state. His list contained 30 species, the identities of some being questionable and the majority unverifiable due to loss of the specimens. Later, Gaufin (1956) published on southwestern Ohio, bringing to 53 the number of species known from the state. His specimens were mainly larvae, but his material exists in various collections, especially at the Monte L. Bean Museum at Brigham Young University (BYUC) and in the Illinois Natural History Survey Insect Collection (INHS). Tkac (1979) conducted a more comprehensive study across the northeastern quarter of the state, but producing only 54 species. His dissertation included the first illustrated taxonomic key to Ohio stonefly larvae and adults. Relatively few of Tkac's specimens have been located and Dr. Ben Foote (pers. comm.) confirms that they are not at Kent State University where the degree was conferred. Late in the current study it was suggested that specimens may reside in the United States National Museum (USNM), but no formal records indicate such a donation ever took place. Many additional studies of a narrower scope have been published, either documenting the stonefly fauna of single streams, as taxonomic revisions, or as short updates to the known fauna. All known works have been documented and discrepancies in name usage have been reconciled in this document.

A much needed update of the Ohio fauna was begun in the 1980s and continued through the 1990s, conducted by RWB, SMC, BJA, and Ralph F. Kirchner (Wheeling, West Virginia). These efforts did not result in publication, but their thousands of specimens form the basis of this work. Beginning in 2005, RED and SAG borrowed material from individuals and institutions, identified the specimens, digitized the label data for 4,080 vials and pins of stoneflies, and georeferenced all locations, resulting in DeWalt et al. (2012). Subsequently, Grubbs et al. (2013b) discussed the distribution of some uncommon and rare species occurring in Ohio, but reported no additional species. Since then, a large collection of additional Ohio stoneflies was donated to the INHS by the Ohio Biological Survey. In addition, many more Ohio Environmental Protection Agency (OEPA) records were made available that dramatically improved the coverage of several species and underrepresented drainages.

Other specimens that improved our coverage include a substantial number of records from Edge of Appalachia Preserve (Adams County, Ohio Brush Creek drainage) collected by RED and specimens collected by Gary A. Coovert since 2004 from Crane Hollow Nature Preserve (Hocking County, Queer Creek drainage). Both locations added new locations for several rare species and confirmed the presence of another. All total, 7,723 specimen records now exist for Ohio stoneflies. This dramatic increase in specimens makes an update desirable, provides an opportunity to present a complete historical accounting of stonefly research conducted in Ohio, explore some relationships of species richness to drainage characteristics, add range maps, conduct analyses of stream widths used by species, and present an analysis of the succession of adult presence throughout the year. None of these analyses were present in DeWalt et al. (2012), though some distribution maps for rare species were provided in Grubbs et al. (2013b).

This publication is volume II in a series of atlases of aquatic insects inhabiting Ohio and complements volume I on caddisflies (Armitage et al. 2011). Future volumes will provide information on Ohio mayflies, aquatic beetles, crane flies, and aquatic and semiaquatic Heteroptera.

## Materials and Methods

**Digitization of specimen data.** Data presented in this work represents a combination of verified specimens, specimen data from the OEPA, and trusted literature. We verified identifications of many of the most difficult to identify species among the OEPA specimens, strongly supporting their inclusion in this study. The specimen data source and number of records (# of vials or pins) are provided for each institution and colleague who provided specimens/data. The methodology for preparing specimens is available in DeWalt et al. (2012). We associated most specimens with their database record using a paper catalog number—a unique identifier. Unfortunately, this was not the case for OEPA specimens, the Western Kentucky University material, and literature sources. Specimen data were gathered in accordance with iDigBio (2014a) wet collection protocols. All data will be shared with the Global Biodiversity Information Facility (GBIF) and with iDigBio (2014b).

Most location labels printed prior to 2000 did not contain geographic coordinates. We georeferenced these locations using Acme Mapper 2.1 (Acme Mapper 2016, datum WGS-84). In the USA, this program provides topographic, satellite, and road map coverages that ensure the greatest possibility of finding complex locations. In addition, where collectors provided coordinates they were projected to verify that the coordinates matched verbal descriptions (correct county, distance and direction from locality, road crossing). Where they did not match, coordinates were corrected or recorded with lower precision in the database. We used a decimal degree format, most often to five significant figures, to improve the usability of the data by others. Estimated precision is presented as a radius in meters. Maps were exported from an ArcView 9.3 (ESRI) project file using a WGS-84 projection, overlaid on United States Geological Survey Hierarchical Unit Code eight (USGS HUC8, 42 drainages) scale drainages with outlines of the 88 Ohio counties. A map was constructed with all unique locations, and individual maps for each species.

**Succession of species.** Adults of stonefly species succeed each other as they emerge throughout the year (Stewart and Stark 2002). This is most clearly demonstrated from single site studies (Ernst and Stewart 1985), but regional data may also be used successfully for this type of analysis if latitudinal differences in the data are ignored. Our data are not derived from emergence traps; accordingly, they reflect presence rather than emergence. Adult stoneflies often live one or two weeks past their date of emergence (DeWalt and Stewart 1995). Hence, the succession of adults presented in contains a bias for the presence of adults collected after peak emergence. We have used adult records in the data set to build a table that depicts adult presence throughout the year on a weekly basis. Records for each species were examined and cells in an Excel spreadsheet were shaded corresponding to the intensity of emergence: dark gray when one or more collecting events (site/date combinations) in a week contained ≥3 adults; medium gray when collecting events contained ≤2 adults; and light gray where no adults were present, but when we assumed from larval records and our experience that adults would be available. All outlying dates of emergence were recorded and the species ordered chronologically to display the sequence of emerging species.

**Species richness vs. county and watershed relationships.** All georeferenced specimen records were associated with HUC8 coverage in GIS and the drainage numbers and names were returned to the data. The total species richness and number of unique locations within a HUC8 drainage were compiled. A map depicting of the number of species vs. HUC8 drainage was constructed so that drainages with similar species tallies were similarly color-coded. Scatterplots were constructed of species richness versus HUC8 area in km^2^ and the number of unique locations within a HUC8 to determine if these variables were important to species richness. Deviations from trend lines produced from simple linear regression analyses were noted. Ohio counties, of which there are 88, are geopolitical units for local government (Anonymous 2016). In an effort to determine if there were areas not well sampled across the state, the number of total records were tallied for each county. A histogram was produced that depicts the number of stonefly records for each county. Those counties with high and low richness were examined for where they occurred within the state.

**Distribution of species in stream size/type categories.** Stoneflies live in a wide range of waterbody sizes, even in large lakes. Drainage area and perhaps the number of links (tributaries) are the best measures of stream size and may often be recovered from Geographic Information Systems data layers. However, these data sets often lack data for the smallest streams. To account for this streams were categorize by stream wetted width (1=seep, 2=1-2 m wide stream, 3=3-10 m wide, 4=11-30 m wide, 5=31-60 m wide, 6=>61 m wide, 7=large lake (Lake Erie specifically). These estimates were made from Acme Mapper (2016) satellite coverages using the scale provided by the program. A histogram of the frequency of site/date events within each stream width or lake category was constructed for each species for all sites that could be georeferenced to a stream or lake (91.2% of 7,723 records).

**Access to the data.** All specimen data used in this study are archived as a Darwin Core Archive file supported by Pensoft's Integrated Publishing Toolkit (DeWalt et al. 2016b). This data set contains some duplication in the form of literature records that may also be available as specimen data with unique identifiers, but we included in order to provide a complete record.

## Results

**Figure 1.**
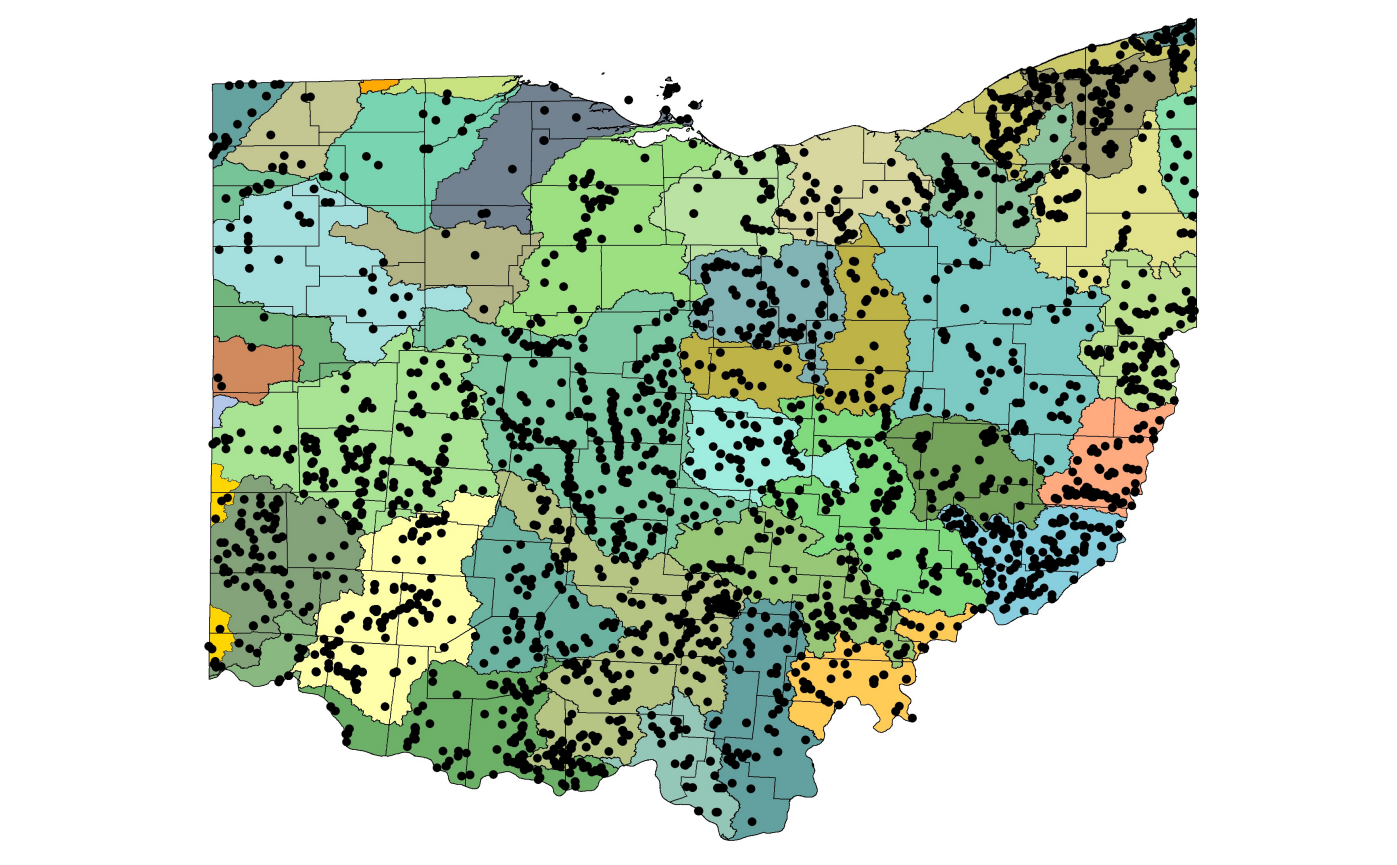
Ohio stonefly collection records, county boundaries, and HUC8 drainages.

A total of 7,797 records were gathered from 21 institutional, government, personal collection sources, and from literature sources (Table 1). Most specimens (>5000) from physical collections were examined by RED & SAG. A total of 2769 unique locations have been georeferenced and mapped (Fig. 1).

At least 53 papers have appeared in print that reference Ohio stoneflies (Suppl. material 1). These include faunal lists and analyses of species richness patterns for the state as a whole or a subset (DeWalt et al. 2012, Gaufin 1956, Grubbs et al. 2013b, Tkac 1979, Walker 1947), records of taxa from a single stream (Beckett 1987, Tkac and Foote 1978, Robertson 1984, Robertson 1979, Fishbeck 1987), discussion of morphological features or genetic diversity for one or more species (Clark 1934, Yasick et al. 2007, Yasick et al. 2015), or included records of Ohio species from descriptions or revisionary or other works (Baumann 1974, Frison 1942, Fullington and Stewart 1980, Grubbs 2006, Grubbs 2015, Grubbs and DeWalt 2008, Grubbs and DeWalt 2012, Grubbs and Stark 2001, Grubbs et al. 2014, Grubbs et al. 2013c, Kondratieff 2004, Kondratieff and Kirchner 1993, Kondratieff and Kirchner 2009, Kondratieff et al. 1988, Nelson 2000, Ricker 1952, Ricker and Ross 1968, Ricker and Ross 1969, Ross and Ricker 1964, Ross and Ricker 1971, Ross and Yamamoto 1967, Ross et al. 1967, Stark 1986, Stark 1989, Stark 2000, Stark 2004, Stark and Baumann 1978, Stark and Baumann 2004, Stark and Gaufin 1974, Stark and Gaufin 1976, Stark and Kondratieff 2010, Stark and Kondratieff 2012, Stark et al. 1988, Stewart 2000, Surdick 2004, Szczytko and Kondratieff 2015, Szczytko and Stewart 1978, Szczytko and Stewart 1981, Young et al. 1989, Zwick 1971).

### Species present and those dismissed from the state tally

In total, 102 species are known to occur in Ohio, though many more names have been associated with the state from previous publications (Table 2, Suppl. material 1). Previous records included *Capnia vernalis* Newport, 1848 from central Ohio (Walker 1947) and repeated by Gaufin (1956). No specimens exist in the collections of museums visited by the authors (Table 1). This species is generally more northern in distribution (DeWalt and South 2015) and is dismissed from occurrence in Ohio. Tkac (1979) lists *Leuctra monticola* Hanson, 1941 from the state. This is undoubtedly a misidentification of *Leuctra alexanderi* Hanson, 1941. *Taenionema atlanticum* Ricker & Ross, 1975 was listed for Ohio by Stewart and Stark (2002). This is an error and the species is removed from the Ohio list.

Larvae of the *Pteronarcys scotti* Ricker, 1952 species group, what was once considered the subgenus *Allonarcys* Needham & Claassen, 1925, have spine-like, paired lateral projections on each abdominal segment (Stark and Szczytko 1982). Bolton (2010) recently reported from Ohio larvae of a *Pteronarcys* with lateral abdominal projections (e.g., P. cf. biloba Newman, 1838), though Tkac (1979) was the first to report it. No adults of this species have been collected despite repeated attempts to locate them in their Lake and Ashtabula county streams (RED and Donald Dean of Ohio State University have searched). Three authors have placed *P. pictetii* Hagen, 1873 as resident in Ohio (Gaufin 1956, Nelson 2000, Stewart and Stark 2002). Gaufin's records are of larvae that others have simply taken for granted. The only *Pteronarcys* species confirmed from an adult, from a single female specimen, is that of *P. dorsata* (Say, 1823) (DeWalt et al. 2012).

Tkac (1979) lists *Alloperla neglecta* Frison, 1935 from Ohio, but no specimens have been recovered and illustrations in his dissertation could represent other species. The epiprocts of *A. neglecta* and *A. concolor* Ricker, 1936 are similar (Kondratieff and Kirchner 1993, Surdick 2004). We maintain this species on the list, but are uncertain of its validity. *Sweltsa mediana* Banks has been reported for Ohio by several authors (Walker 1947, Fishbeck 1987, Gaufin 1956, Tkac 1979, Tkac and Foote 1978). These are all undoubtedly referable to the recently described *Sweltsa hoffmani* Kondratieff & Kirchner, 2009 as are *S. onkos* (Ricker, 1936) listed for Ohio by Stewart and Stark (2002).

DeWalt et al. (2012) listed *Acroneuria kirchneri* Stark & Kondratieff, 2004 from Ohio on the basis of several females; however, these have recently been re-examined by Boris C. Kondratieff and found to be *A. kosztarabi* Kondratieff & Kirchner, 1993. Several authors have listed *Neoperla clymene* (Newman, 1839) from Ohio (DeWalt et al. 2012, Gaufin 1956, Needham and Claassen 1925, Walker 1947, Tkac 1979), these are all from larval collections and could be any one of seven species known from Ohio. We remove this species from the Ohio list.

Stewart and Stark (2002) and Stark (2004) listed *Perlesta cinctipes* (Banks, 1905) from Ohio. We believe that *P. cinctipes* does not occur in the state, but that specimens named as such represent a new, darkly colored species that we have provisionally named *Perlesta* I-4. This species occurs in Ohio, Indiana, and Kentucky and SAG & RED are in the process of describing it. *Perlesta nitida* Banks, 1948 and *P. lagoi* Stark, 1989 are nearly identical as adults and both have been listed from the state (DeWalt et al. 2012, Grubbs and Stark 2001). We have opted to use *P. lagoi* at this point to represent all the medium-to-small sized *Perlesta* where males have a short caecum with narrow dorsal patch that widens onto the caecum, and where females have a deeply cleft subgenital plate and collarless eggs with fine punctations about the middle. The validity of these two species may require a large series and much genetic work to determine. DeWalt et al. (2012) listed *P. golconda* DeWalt & Stark, 1998 from the state, but this has turned out to be a database error. This species has been removed from the Ohio list.

Three publications listed *Isoperla namata* Frison, 1942 from Ohio (Stewart and Stark 2002, Tkac 1979, Szczytko and Kondratieff 2015). We believe that all specimens previously identified as *I. namata* are *I. montana* (Banks), though an outside possibility exists that at least some of these are *I. kirchneri* Szczytko & Kondratieff, 2015. The current work confirms the presence of *I. orata* Frison, 1942 from Crane Hollow Nature Preserve in Hocking County. Additionally, a new state record of *Isoperla richardsoni* Frison, 1935 was confirmed from the Ohio River in Adams County. This year, one female of *Malirekus iroquois* Stark & Szczytko, 1988 was reared from Little Lyons Creek in Ashland County, Ohio. This confirms the presence of *M. iroquois* in Ohio.

Tkac (1979) reported *Cultus decisus* (Walker, 1852) from northeastern Ohio. This was prior to the Stark et al. (1988) re-examination of eastern North American *Cultus*. The revision created a more northern nominotypical subspecies, *C. decisus decisus* (Walker, 1852) and the more southerly distributed *C. decisus isolatus* (Banks, 1920). They also confirmed the validity of *C. verticalis* (Banks, 1920). Although Tkac provided drawings of the one male he collected, it is impossible to ascertain whether the specimen was *C. verticalis*, *C. d. decisus*, or *C. d. isolatus*. Fresh specimens are needed to make this determination. We will retain *C. decisus* in the list of Ohio species until resolution of this conundrum is possible.

### Species richness vs. watershed and county relationships

**Figure 2.**
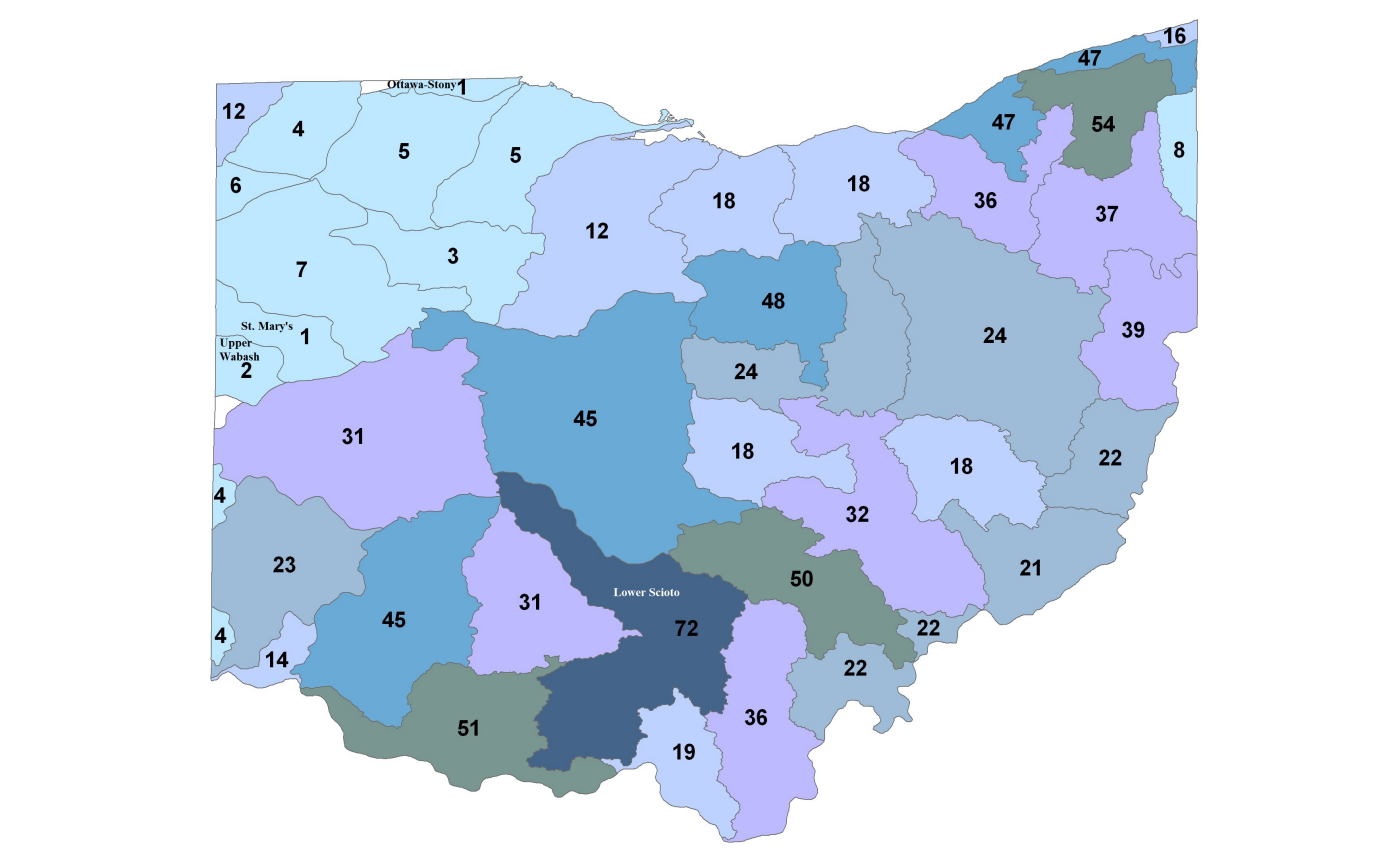
Stonefly species richness for 41 Ohio USGS HUC8 watersheds. Watershed color coded by similar richness. Watershed names for some species poor and species rich drainages provided.

Stonefly species richness varied tremendously with HUC8 affiliation (Fig. 2). The Lower Scioto River drainage supported 72 stonefly species, 18 more than the next richest drainage. The richest drainages were in northeast, south-central, and southern Ohio. These areas are heavily forested, have the highest slopes (DeWalt et al. 2012), and were part of or adjacent to the unglaciated, Western Allegheny Plateau of Ohio . The drainages with the lowest richness were mostly found in the northwestern quarter of Ohio, which was the most glaciated area of Ohio and site of the Great Black Swamp during the post-glacial period. Eight western drainages supported five or fewer species with three drainages, the Upper Wabash, Ottawa-Stony, and St. Mary's supporting only one or two species (Fig. 2). Dominated by glacial lake plain topography, these drainages have low slope values, fine-grained sediments, and now, approximately 90% coverage in row crop agriculture (DeWalt et al. 2012). Historically, they would not have supported many stonefly species, and with the agriculturally modified landscape, few remain.

**Figure 3.**
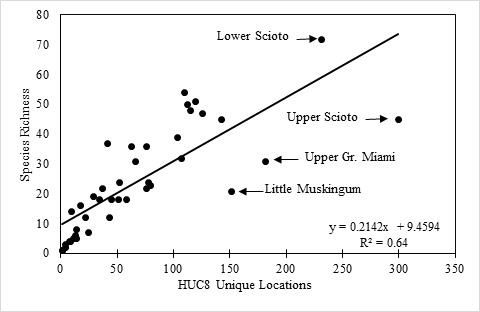
Stonefly species richness vs. HUC8 surface area (km^2^). Simple linear regression equation, R^2^, and line-of-best-fit provided. Lower Scioto watershed point indicated.

**Figure 4.**
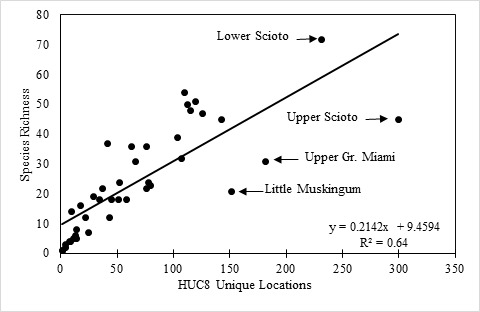
Stonefly species richness vs. number of HUC8 unique locations. Simple linear regression equation and R^2^ provided. Names of HUC8s with greatest deviation from line-of-best-fit provided.

Surface area of HUC8 drainages appears to be an unimportant predictor of stonefly species richness (Fig. 3). One point is well above the line-of-best-fit, that of the Lower Scioto drainage. It is the richest, despite not being the largest, HUC8 drainage. Many relatively small HUC8s have high richness, while many intermediate sized drainages support only a few stonefly species. The number of unique locations sampled within a watershed appears to be a much stronger predictor of stonefly species richness (Fig. 4). Again, the Lower Scioto drainage exceeds predictions. Conversely, the Upper Scioto, the Upper Greater Miami, and Little Muskingum drainages all fall below the line-of-best-fit. These drainages are either largely agricultural, have high industrialization, or have large human populations in them, all conditions that would lead to lower than expected stonefly richness.

**Figure 5.**
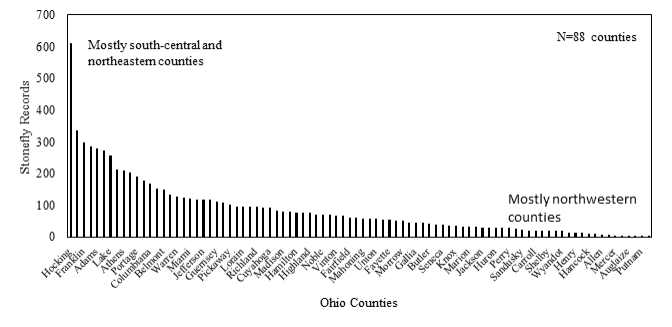
Stonefly species richness for 88 Ohio counties (only every other name presented). Regions of the state with richest and poorest totals presented.

At least one stonefly record is available for each of Ohio's 88 counties (Fig. 5). Hocking County in south-central Ohio has more stonefly records than any other county by nearly a factor of two. It is the most important county contributing to the richness of the Lower Scioto drainage (59 of 72 spp., next has 44 spp.). Because Hocking County has never been glaciated, it maintains a rugged topography with deep ravines composed of Pennsylvanian and Mississippian age sandstones and shales, respectively (Hansen 1975). These ravines and the creation of Ohio State Forests in 1915 protected streams from logging and farming, preserving much of the rich native stonefly fauna of the area. Protected areas in the county include Hocking Hills State Park, Hocking Hills State Forest, and the small but species-rich Crane Hollow Nature Preserve. Other species rich counties are located in northeastern, south-central, and southern Ohio. Those counties with the lowest diversity are generally northwestern, again their diversity suffering from historically flat terrain, lake plain topography, sluggish streams, and the contemporary dominance of agricultural land use (DeWalt et al. 2012).

### Succession of adult presence

Ohio stonefly adults may be obtained in nearly every month of the year, but are most frequently collected from January to July (Table 3). Adult phenology expresses a strong phylogenetic component in that the superfamily Nemouroidea (Capniidae, Taeniopterygidae, Nemouridae, and Leuctridae) emerge earliest in the year. Indeed, Capniidae and Taeniopterygidae and subsets of the other two families are generally referred to as "winter stoneflies" due to their emergence as adults in winter. There is often a short lull in adult presence in mid-April before other species of leuctrids and nemourids appear. Most of the remainder of superfamily emerge in spring and early summer, but *Leuctra tenuis* (Pictet, 1841) persists well into autumn.

The superfamilies Perloidea (Chloroperlidae, Perlidae, Perlodidae) and Pteronarcyoidea (Peltoperlidae, Pteronarcyidae) contain spring and summer emerging species. Chloroperlidae, such as *Sweltsa hoffmani* Kondratieff & Kirchner, 2009, often begin emerging in late April; other "sallflies" follow through early July. Perlodidae are commonly known as "spring stoneflies" since most of their members emerge before summer. *Isoperla bilineata* (Say, 1823) is the earliest emerging perlodid species with some records beginning in late March, particularly from larger rivers in the southern part of the state. The rest of the species in the family are present primarily in May and early June. Adult presence of *I. signata* (Banks, 1902) and *I. transmarina* (Newman, 1838) is inferred (see light gray of Table 3) from larval records and regional experience since no adults were collected for these species.

Perlidae adults are present from early spring until late summer. The females of perlids live a comparatively long life, hence their adult presence spans up to three months for some species. The single Peltoperlidae species, the roachfly *Peltoperla arcuata* Needham, 1905, is present in late May through mid-June. The adult presence of Pteronarcyidae, or salmonflies, in Ohio is rather a mystery since only a single adult of one species, *Pteronarcys dorsata* (Say, 1823), has been collected. The adult presence of P. cf. biloba Newman, 1838 is inferred from larval records and professional judgement.

The bias in this data set for the protracted presence of spent (all or most eggs expelled, but still alive) females should be accounted for by future researchers of stonefly adults. Consulting the dataset associated with this work will improve a researcher's ability to find adult stoneflies. Paying particular attention to whether a year is above or below average in air temperature is also important, as will be future changes in climate that shift emergence of all species to earlier weeks. Some shifting has already undoubtedly occurred.

### Species distributions, stream size affiliation, and Adult Presence Phenology

**Figure 6.**
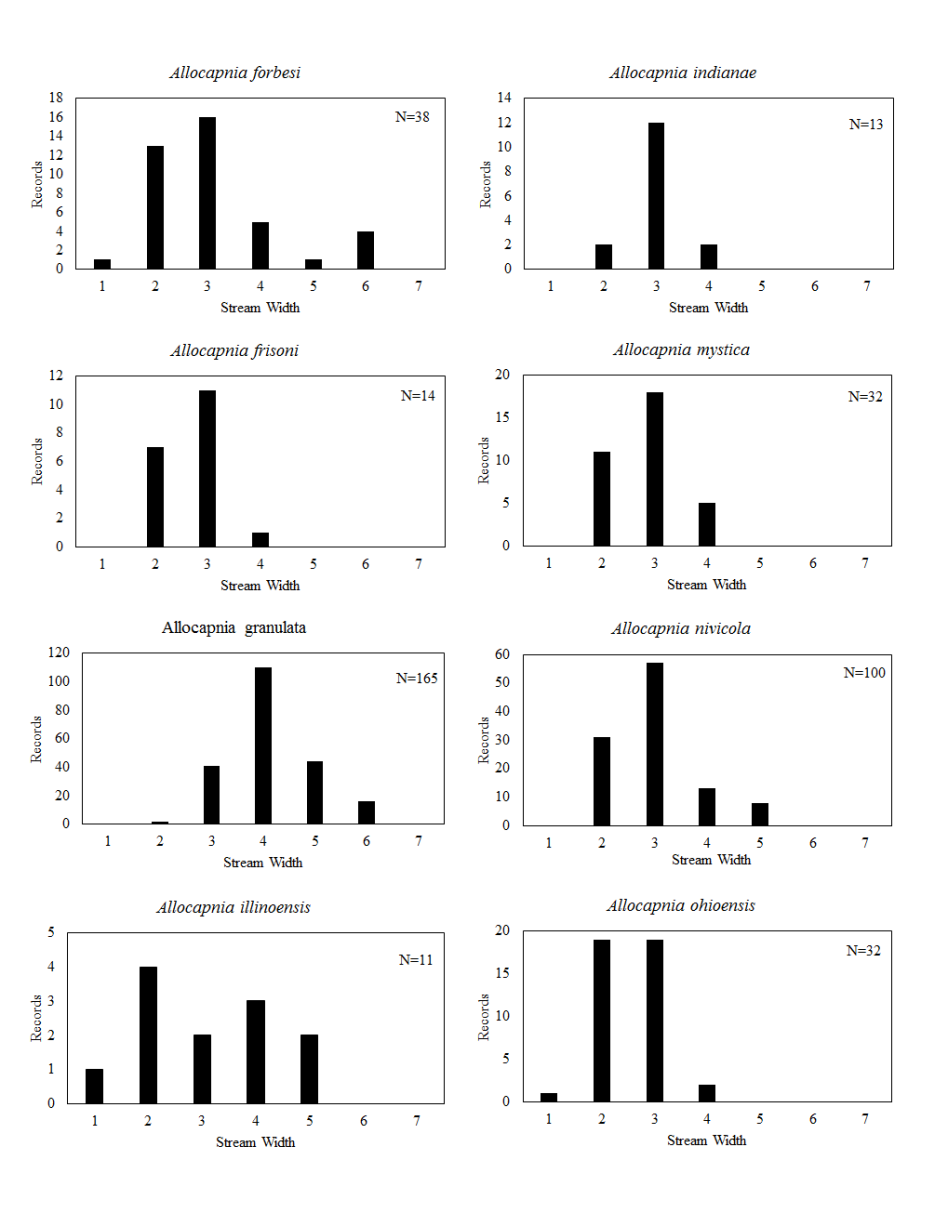
Stream width categories occupied by Capniidae species in Ohio. Stream width categories: 1=seep, 2=1-2 m, 3=3-10 m, 4=11=30 m, 5=31-60 m, 6=>60 m, 7=Lake Erie.

**Figure 7.**
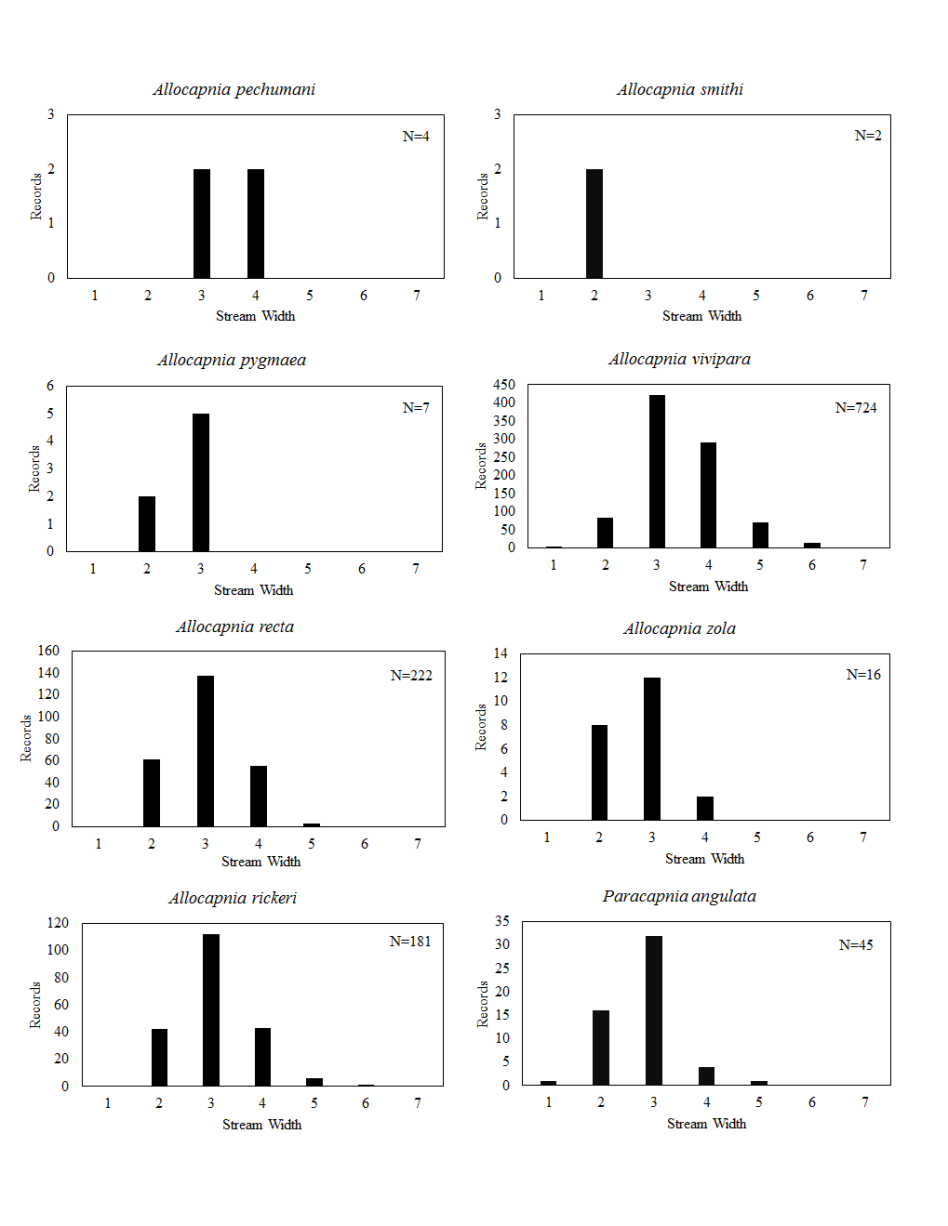
Stream width categories occupied by Capniidae species in Ohio. Stream width categories: 1=seep, 2=1-2 m, 3=3-10 m, 4=11=30 m, 5=31-60 m, 6=>60 m, 7=Lake Erie.

**Figure 8.**
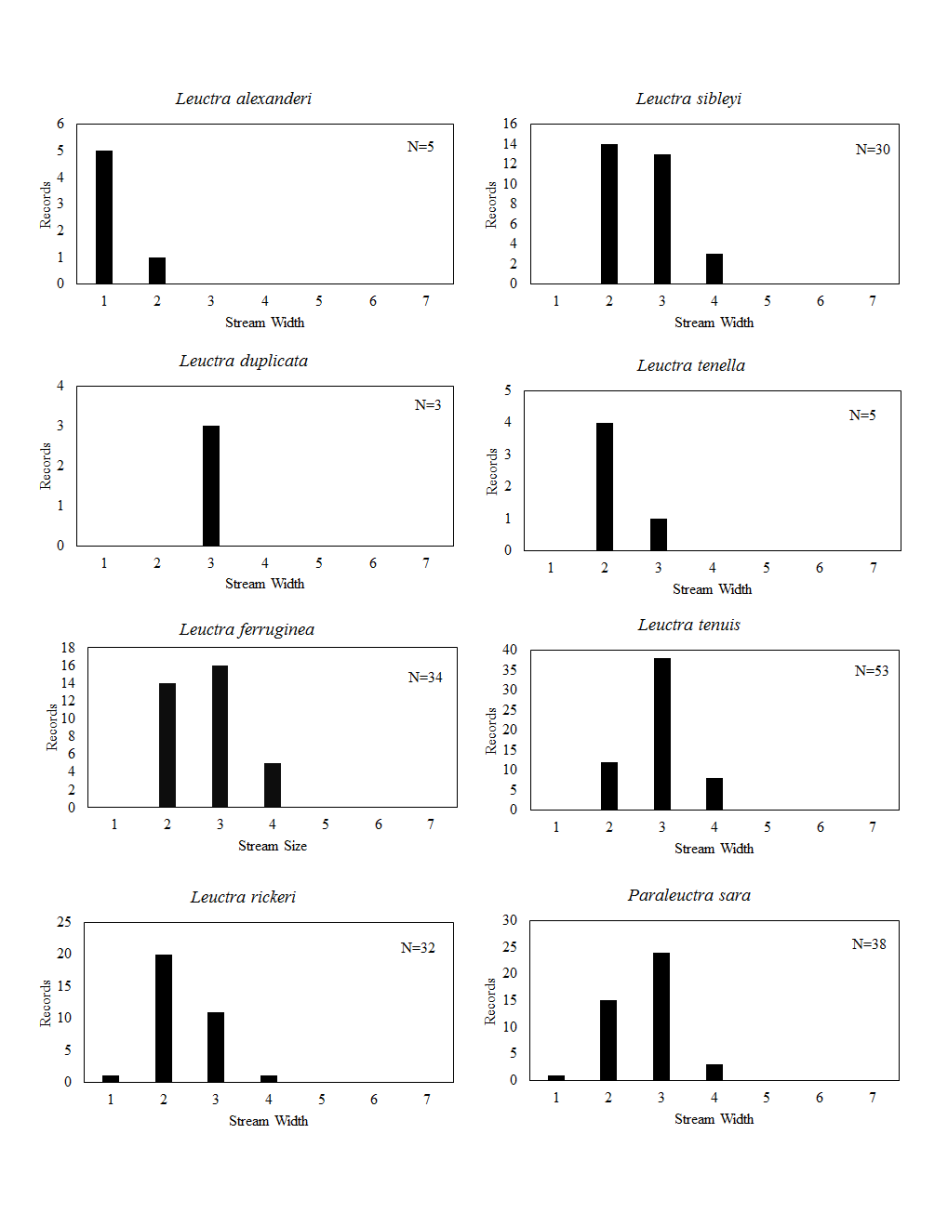
Stream width categories occupied by Leuctridae species in Ohio. Stream width categories: 1=seep, 2=1-2 m, 3=3-10 m, 4=11=30 m, 5=31-60 m, 6=>60 m, 7=Lake Erie.

**Figure 9.**
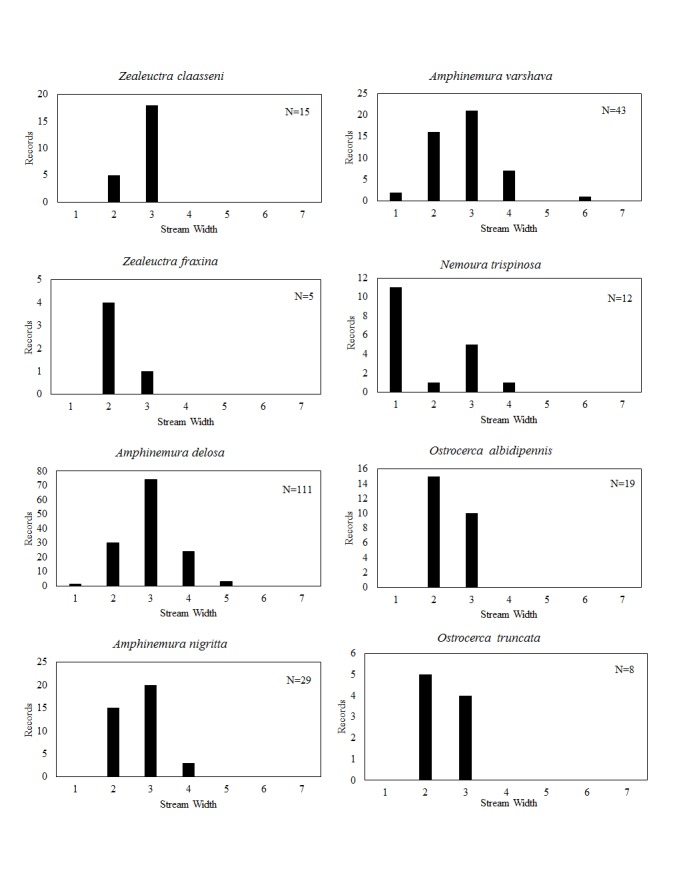
Stream width categories occupied by Leuctridae and Nemouridae species in Ohio. Stream width categories: 1=seep, 2=1-2 m, 3=3-10 m, 4=11=30 m, 5=31-60 m, 6=>60 m, 7=Lake Erie.

**Figure 10.**
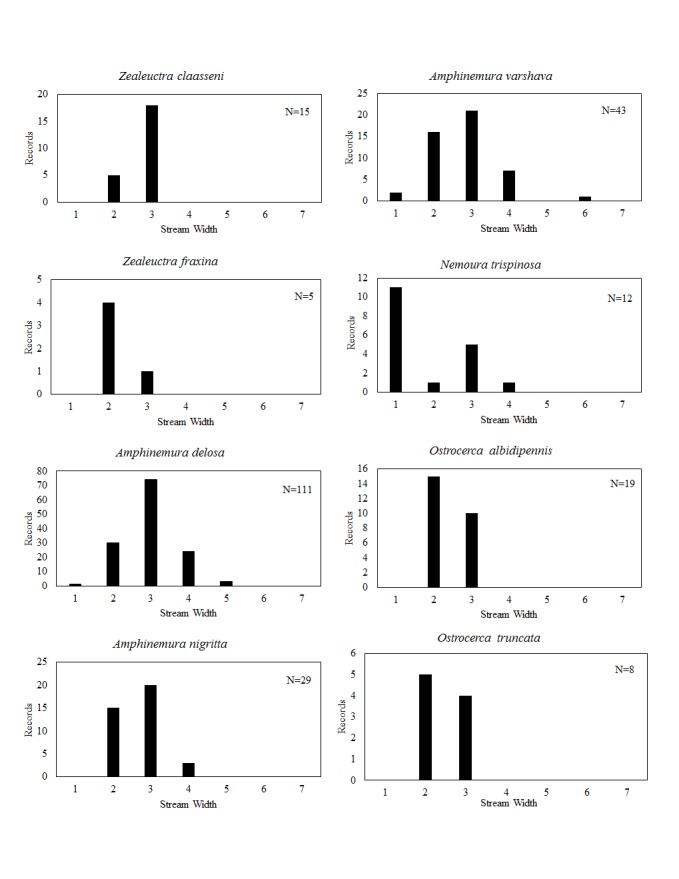
Stream width categories occupied by Nemouridae and Taeniopterygidae species in Ohio. Stream width categories: 1=seep, 2=1-2 m, 3=3-10 m, 4=11=30 m, 5=31-60 m, 6=>60 m, 7=Lake Erie.

This section documents the relative stream size occupied (Figs 6, 7, 8, 9, 10, 12, 13, 14, 15, 16, 17, 18), the distribution of the species (Figs 19, 20, 21, 22, 23, 24, 25, 26, 27, 28, 29, 30, 31), and the adult presence phenology (Table 3) of each stonefly species found in Ohio. Family names occur in phylogenetic order, while genus and species names are alphabetized. Range wide discussion of distributions originate from Plecoptera Species File (DeWalt et al. 2016a), this citation being used only in this paragraph to reduce repetition in succeeding text. General distributions are occasionally supplemented with citations from other recent treatments. Distributions are discussed in terms of the following: Interior Highlands (Ozark and Ouachita mountains of Arkansas, Missouri, and Oklahoma), Appalachian Mountains, glaciated vs unglaciated landscapes, Atlantic Coast, USA states, and Canadian provinces. Each taxon name is easily queried from Plecoptera Species File (DeWalt et al. 2016a), resulting in a species page with abundant nomenclatural, taxonomic, and distributional information.

### Capniidae. Snowflies

*Allocapnia forbesi* Frison, 1929. This species is collected most frequently in headwater streams, but may be taken from larger ones as well (Fig. 6). Its Ohio distribution encompasses the unglaciated southern half of the state with few outliers (Fig. 19). We expect many more records from small, clear streams in the unglaciated hills of southern Ohio. Adult presence spans December to March with spent females still being available into April (Table 3). *Allocapnia forbesi* inhabits the Ohio River Valley from Illinois to West Virginia.

*Allocapnia frisoni* Ross & Ricker, 1964. This species occurs in headwater streams (Fig. 6), mainly in the south-central region of the state (Fig. 19). Adults are present from late December through March (Table 3). Collecting efforts in southeastern Ohio should produce additional records. Overall, *Allocapnia frisoni* is an Appalachian-distributed species known from Kentucky and Tennessee to Virginia and northeast to Pennsylvanian and New York.

*Allocapnia granulata* (Claassen, 1924). This species inhabits comparatively larger streams and rivers than *A. forbesi* and *A. frisoni* (Fig. 6) and is relatively common throughout the state (Fig. 19). Adults occur from January through March (Table 3). *Allocapnia granulata* occupy streams from Oklahoma and Texas eastward into Quebec.

*Allocapnia illinoensis* Frison, 1935. This uncommon species lives in small streams (Fig. 6) in the eastern half of the state (Fig. 19). Adults occur from January through March (Table 3). The species inhabits mainly glaciated landscapes in eastern North America.

*Allocapnia indianae* Ricker, 1952. This species occupies small streams (Fig. 6) in the unglaciated south-central region of Ohio (Fig. 19). Adults are present from late January through March (Table 3). This species inhabits small Ohio River Valley streams from Indiana east to West Virginia with additional adjunct populations in New York.

*Allocapnia mystica* Frison, 1929. This species occurs mainly in small streams (Fig. 6) in the southern half of the state (Fig. 19). Adults emerge in January and persist through March (Table 3). This is a common species of unglaciated landscapes from Arkansas and Missouri eastward to Virginia.

*Allocapnia nivicola* (Fitch, 1847). This common species occupies a broad range of stream sizes (Fig. 6) across all but the northwest corner of the state (Fig. 19). Adults emerge mainly in January but persist through March (Table 3). This species inhabits much of the deciduous forest of eastern North America.

*Allocapnia ohioensis* Ross & Ricker, 1964. This species occurs mostly in small streams (Fig. 6) in the unglaciated southern half of the state (Fig. 19). Adults emerge in January and may be present through early April (Table 3). The range of *A. indianae* encompasses the unglaciated portion of the Ohio River Valley from Indiana east to West Virginia with disjunct populations in New York.

*Allocapnia pechumani* Ross & Ricker, 1964. Our records demonstrate this rare species to inhabit medium sized streams (Fig. 7) in the glaciated northeastern corner of the state (Fig. 20). Adults occur during February and March (Table 3). *Allocapnia pechumani* is also known from Pennsylvania northeast to New Brunswick.

*Allocapnia pygmaea* (Burmeister, 1839). This species occurs in seven small streams (Fig. 7) in southern and northeastern Ohio (Fig. 20). Our data suggest a mid-February through March emergence (Table 3). *Allocapnia pygmaea* occurs over much of eastern North America.

*Allocapnia recta* (Claassen, 1924). This species inhabits small streams (Fig. 7) across most of the state (Fig. 20). DeWalt et al. (2005) and DeWalt and Grubbs (2011) also report it from some of the largest streams in Illinois and Indiana, respectively. This is the earliest emerging snowfly, collected as early as mid-November, but continuing through March (Table 3). *Allocapnia recta* occurs throughout much of eastern North America west of the Mississippi River.

*Allocapnia rickeri* Frison, 1942. This species inhabits small streams (Fig. 7) across most of the state (Fig. 20). Adult presence encompasses January through April (Table 3). The species occurs widely across eastern North America.

*Allocapnia smithi* Ross & Ricker, 1971. This is one of the rarest stonefly species inhabiting eastern North America. One male and one female are known from two small ravine streams in Warren County (Figs 7, 20). Both specimens were collected in mid-February (Table 3). *Allocapnia smithi* is restricted to unglaciated regions of Illinois, Indiana and Ohio, and in both Kentucky and central Alabama.

*Allocapnia vivipara* (Claassen, 1924). This species occurs in a broad range of stream sizes (Fig. 7) across all of Ohio (Fig. 20). Adults emerge as early as mid-December, persisting through April (Table 3). *Allocapnia vivipara* exhibits the widest distribution of any *Allocapnia* in eastern North America.

*Allocapnia zola* Ricker, 1952. This species occurs in small streams (Fig. 7) in three adjacent counties of the Hocking Hills region of southern Ohio (Fig. 20). Adults occur from January through April (Table 3). Overall, *A. zola* ranges from Ohio to Appalachian Kentucky, northeastward to New Brunswick.

*Paracapnia angulata* Hanson, 1961. This species inhabits mainly small, cold streams (Fig. 7), exhibiting a broad, yet patchy distribution across the state (Fig. 20). New records from the tributaries of Ohio Brush Creek, Edge of Appalachia Preserve, in Adams County suggest that the species is more widely distributed in spring fed streams of southwestern Ohio than currently known. Depending upon latitude, adults emerge in January, persisting through April (Table 3). This species is extensively distributed across eastern North America.

### Leuctridae. Needleflies

*Leuctra alexanderi* Hanson, 1941. This species is rare, occurring in only three small streams (Fig. 8) in the eastern half of the state (Fig. 21). Adult presence extends from mid-May through mid-June (Table 3). The distribution of this species encompasses the central and southern Appalachian Mountains from Tennessee north to Pennsylvania, into eastern Ohio.

*Leuctra duplicata* Claassen, 1923. This species occurs in two small (Fig. 8), closely adjacent streams in Ashtabula County (Fig. 21). Adults occur in early June (Table 3). This species is likely more abundant in northeastern Ohio than our data suggest. A mainly Appalachian-distributed species, it occurs from Virginia northeast through eastern Canada.

*Leuctra ferruginea* (Walker, 1852). This species occurs in small streams (Fig. 8) in the eastern half of the state (Fig. 21). Adult presence lasts from mid-May through July (Table 3). *Leuctra ferruginea* inhabits small streams across much of eastern North America.

*Leuctra rickeri* James, 1976. This species is extremely common in the south-central region of the state (Fig. 21) where it inhabits mainly small streams (Fig. 8). Adult presence extends from late May through early July (Table 3). We believe that this species must occur in southwestern Ohio, though it has not been collected there. It occurs in the adjacent Indiana tributaries of the Whitewater River (DeWalt and Grubbs 2011). This species occurs from the Florida Panhandle north to Iowa, east to Michigan and Maryland.

*Leuctra sibleyi* Claassen, 1923. This species occurs in small streams (Fig. 8) in the southern and eastern halves of the state (Fig. 21). Adults begin emergence in early March and are present until mid-June (Table 3). This species is broadly-distributed east of the Mississippi River in north of Alabama.

*Leuctra tenella* Provancher, 1878. This species resides in small streams (Fig. 8) in the Hocking Hills region of south-central Ohio (Fig. 21). Adults are present from late May through early June (Table 3). This species is a broadly-distributed Appalachian species known from North Carolina northeast to the Canadian Maritime Provinces. Doubtful records exist for Minnesota and Wisconsin.

*Leuctra tenuis* (Pictet, 1841). This species is most prevalent in small streams (Fig. 8) and exhibits a scattered distribution throughout much of the state (Fig. 21). Most records come from the glaciated northeastern region. This is the only predominantly autumn emerging stonefly species in Ohio (Table 3). It occupies small upland streams and springs from the Interior Highlands of Oklahoma, Arkansas, and Missouri eastward and northward to the Maritime Provinces of Canada.

*Paraleuctra sara* (Claassen, 1937). This species occurs in smaller streams (Fig. 8) in the eastern and southern halves of the state (Fig. 21). Adult collections center on March and April (Table 3). This is a broadly-distributed Appalachian species known from Alabama northeast to the Canadian Maritime Provinces.

*Zealeuctra claasseni* (Frison, 1929). Collections are from small streams (Fig. 9) primarily from the unglaciated southern half of the state (Fig. 22). Adults occur appear in March and April (Table 3). This species generally inhabits unglaciated landscapes from Texas north to Kansas and east to West Virginia.

*Zealeuctra fraxina*. This rarely collected species inhabits headwater streams (Fig. 9) in the south-central region of the state (Fig. 22). Adult presence spans February through March (Table 3). Collecting intermittent streams of southern Ohio in February should produce additional records. This species occurs only east of the Mississippi River in unglaciated landscapes from Illinois to Virginia.

### Nemouridae. Forestflies

​*Amphinemura delosa* (Ricker, 1952). This common species inhabits a broad range of stream sizes (Fig. 9) across most of the state (Fig. 22). Mid-April through July encompasses its flight period (Table 3). The distribution of this species spans much of eastern North America.

*Amphinemura nigritta* (Provancher, 1876). This species inhabits small streams (Fig. 9) and is much less common than *A. delosa*. It occurs across the eastern half of the state (Fig. 22). Adults occur mostly in May through late July (Table 3). *Amphinemura nigritta* occurs over nearly all of eastern North America.

*Amphinemura varshava* (Ricker, 1952). This species inhabits a broad range of stream sizes in Ohio (Fig. 9), occurring mainly in the southern half of the state (Fig. 22). Adult presence spans late April through June (Table 3). *Amphinemura varshava* occurs in a narrow area from Wisconsin and Iowa south to Kentucky and east through Ohio.

*Nemoura trispinosa* Claassen, 1923. Several widely-disjunct localities provide habitat for this uncommon species (Fig. 22). This glacial relict has as its southern-most known population in Ohio a series of springs that feed Yellow Springs Creek (Greene County). Adults have been found mainly from mid-April through July (Table 3) and the species most frequently occurs in springs and springbrooks (Fig. 9). This species is distributed from the Canadian Maritime Provinces west to Manitoba and south through previously glaciated landscapes.

*Ostrocerca albidipennis* (Walker, 1852). This headwater species (Fig. 9) primarily inhabits the southern half of the state, but it also occurs in the more northern Mohican State Park area (Ashland County) (Fig. 22). Adults are present mid-April through mid-June (Table 3). *Ostrocerca albidipennis* is known from Michigan east to Ohio and Virginia and northeast to Nova Scotia.

*Ostrocerca truncata* (Claassen, 1923). This is also a headwater species (Fig. 9) occurring mainly in the Hocking Hills region of southern Ohio (Fig. 22). One literature record (Tkac 1979) places the species in Stebbins Gulch (Geauga County). Adults fly mid-April through May (Table 3). Collecting efforts in headwater streams of southern and eastern Ohio should produce additional records. The species is broadly-distributed small, woodland streams from Indiana and Kentucky east to Virginia and north to Quebec.

*Prostoia completa* (Walker, 1852). This species is rarely collected in Ohio, though we believe it should be more abundant (Fig. 23). The few specimens known originate from headwater to mid-order streams (Fig. 10). Adults occur from March and April (Table 3). The species occurs extensively across eastern North America.

*Prostoia similis* (Hagen, 1861). This species is more widely distributed in Ohio and more abundant where found than *P. completa* (Fig. 23). It too inhabits small streams (Fig. 10). Adult presence spans mid-February through May (Table 3). The distribution of *P. similis* in North America is nearly identical to that of *P. completa*.

*Soyedina vallicularia* (Wu, 1923). This common headwater species (Fig. 10) occurs across the state with the exception of the depauperate northwestern counties (Fig. 23). Collecting in perched seeps, springheads, and springbrooks will undoubtedly result in additional records. Larvae and adults may be collected from wooded seepage areas even where there is little perceptible flow. Adults occur from January through mid-June (Table 3). *Soyedina vallicularia* inhabits springs and springbrooks from Iowa eastward to the Atlantic Coast and from Tennessee northward to the eastern Canadian provinces.

### Taeniopterygidae. Willowflies

*Strophopteryx fasciata* (Burmeister, 1839). This species inhabits larger streams and rivers (Fig. 10). Although collections cluster in the southwestern quarter of the state (Fig. 23), this species should be more widely distributed. Adults occur from mid-February through mid-April (Table 3). The species ranges broadly across eastern North America.

*Taeniopteryx burksi* Ricker & Ross, 1968. This species inhabits a large range of stream sizes (Fig. 10). A predictable outcome of this is that the species has one of the broadest distributions for Ohio stoneflies (Fig. 23). Adult presence spans January through mid-April (Table 3). *Taeniopteryx burksi* occurs in nearly every state east of the Rocky Mountains.

*Taeniopteryx lita* Frison, 1942. Adults of this species have yet to be collected in Ohio, the sole specimen being a mature larva taken from the Ohio River in southeastern Ohio (Figs 10, 23). Adult presence probably encompasses the same weeks in late winter as other *Taeniopteryx* species (Table 3). This species is known from unglaciated landscapes from Texas east to Florida and north to Illinois, Indiana, Ohio, and New Jersey.

*Taeniopteryx maura* (Pictet, 1841). Large streams and small rivers support this species in Ohio (Fig. 10) and its distribution is of a scattered nature, being found in all corners of the state except the northwest (Fig. 23). Adult presence spans from January through March (Table 3). This species appears to be most broadly distributed in unglaciated regions of eastern North America from Texas to Maine.

*Taeniopteryx metequi* Ricker & Ross, 1968. This species typically inhabits smaller streams and rivers (Fig. 10) in the northeastern and southern regions of the state (Fig. 23). Adult presence spans from January through March (Table 3). *Taeniopteryx metequi* is distributed mainly in unglaciated landscapes from the Interior Highlands eastward to North Carolina, an isolated population from Alabama, and then into southern Ontario.

**Figure 11.**
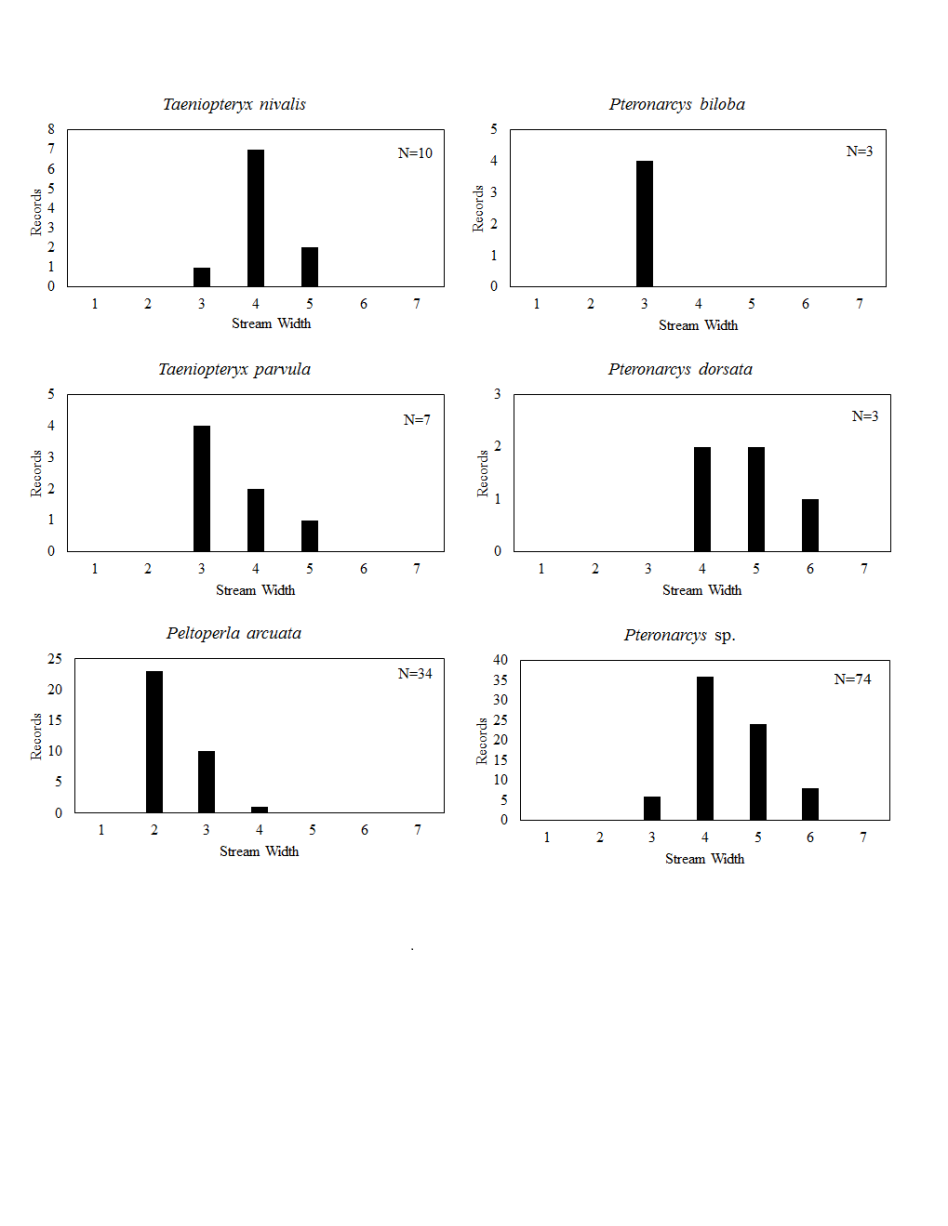
Stream width categories occupied by Taeniopterygidae, Peltoperlidae, and Pteronarcyidae species in Ohio. Stream width categories: 1=seep, 2=1-2 m, 3=3-10 m, 4=11=30 m, 5=31-60 m, 6=>60 m, 7=Lake Erie.

*Taeniopteryx nivalis* Fitch, 1847. This species inhabits mid-order streams and small rivers (Fig. 11) in the northern counties of the state (Fig. 24). Adult presence spans February through mid-March (Table 3). *Taeniopteryx nivalis* occurs broadly across the colder regions of North America from Quebec west to California and extends southward to Wisconsin age glacial extent.

*Taeniopteryx parvula* Banks, 1918. This species typically inhabits mid-order streams and small rivers (Fig. 11), its distribution being of a highly scattered nature (Fig. 24). Adult presence spans mid-February through mid-March (Table 3). The distribution of *T. parvula* is extensive, including much of eastern North America and westward to Alberta, Wyoming, Colorado, and New Mexico.

### Peltoperlidae. Roachflies

*Peltoperla arcuata* Needham, 1905. This is the only representative of the family in Ohio. It is a headwater species (Fig. 11), occurring only in the eastern half of the state (Fig. 24). It was once thought to be rare, but OEPA sampling in headwater streams provides several more unique locations. Adults are available from mid-May through early July (Table 3). This species is common in higher gradient streams from Tennessee and Virginia, northeastward to Quebec.

### Pteronarcyidae. Salmonflies

*Pteronarcys cf. biloba* Newman, 1838. The identity of this species is uncertain since no adults have been collected in Ohio. The species occurs in two small streams (Fig. 11) in northeastern Ohio (Fig. 24). This species probably emerges in May (Table 3). This is a broadly-distributed Appalachian species ranging from Alabama and Georgia northeastward to the Canadian Maritime Provinces.

*Pteronarcys dorsata* (Say, 1823). One adult female exists that validates the occurrence of this species in Ohio (Fig. 24). Labels indicate Columbus, 1 May 1906, presumably from the Scioto River (Fig. 11). This is one of the most widely distributed stoneflies in North America.

*Pteronarcys* sp. All *Pteronarcys* larvae inhabiting eastern North America that lack lateral abdominal appendages belong to the *P. dorsata* species group (Stark and Szczytko 1982). *Pteronarcys dorsata* and *P. pictetii* comprise this group--both probably occur in Ohio, given records for Indiana (DeWalt and Grubbs 2011). However, no adults of *P. pictetii* have ever been collected in Ohio. The current school of thought is that the larvae of these two species cannot be reliably separated using any currently known combination of characters. Since there are so few records for the entire genus, we believe it is useful to provide some information for P. *dorsata* group larvae. Most records are for unglaciated or glaciated drainages adjacent to the glacial boundary (Fig. 24). This includes sections of Big Darby Creek, the Clear Fork of the Mohican River, the mainstem and several tributaries of the Walhonding and Kokosing rivers, the Little Beaver Creek drainage, and the Muskingum River. Two additional records are known for the glaciated northeast (East Branch Euclid Creek) and far northwest (St. Joseph River). Larvae of the group have been collected from larger streams up to some of the largest rivers in the state, excepting the Ohio (Fig. 11​).

### Chloroperlidae. Sallflies

**Figure 12.**
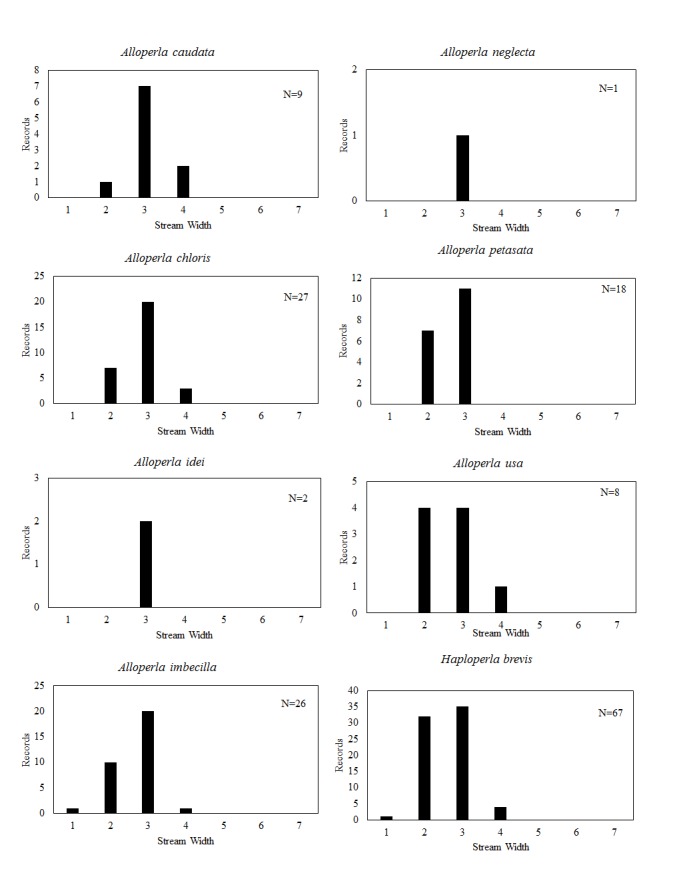
Stream width categories occupied by Chloroperlidae species in Ohio. Stream width categories: 1=seep, 2=1-2 m, 3=3-10 m, 4=11=30 m, 5=31-60 m, 6=>60 m, 7=Lake Erie.

*Alloperla caudata* Frison, 1934. Small to medium sized streams (Fig. 12) in the south-central region of the state support this species (Fig. 25) with adults present in May and June (Table 3). This species is distributed from the Interior Highlands east to Ohio and south to Kentucky, Tennessee, and Alabama.

*Alloperla chloris* Frison, 1934. This too is a small stream *Alloperla* (Fig. 12) that is densely concentrated in tributaries of Lake Erie in northeastern Ohio, but may be found in a few other widely scattered locations (Fig. 25). Adult presence spans May to August (Table 3). This widespread Appalachian species occurs from Georgia northeast to Quebec and New Brunswick.

*Alloperla idei* (Ricker, 1935). This species is rarely collected in Ohio with all three records being assigned to streams between 3 and 10 m width (Fig. 12) in the south-central region of the state (Fig. 25). Adults are present in May (Table 3). This widespread Appalachian species occurs from Georgia northeast to Maine, Ontario, Quebec, and New Brunswick.

*Alloperla imbecilla* (Say, 1823). The species occurs in mainly small streams (Fig. 12) in the south-central and northeastern regions of the state (Fig. 25). Adults fly mainly during May through June (Table 3). This Appalachian species ranges from Kentucky and Virginia north to New York.

*Alloperla neglecta* Frison, 1935. Tkac (1979) reported a single male from Paine Creek in northeastern Ohio (Figs 12, 25). The single adult is from late May (Table 3). Since Tkac's specimen has not been located, some uncertainty continues to exist about this record (DeWalt et al. 2012, Grubbs et al. 2013b). The confirmed distribution of this species encompasses the southern Appalachian Mountains in North Carolina, Tennessee, and Virginia.

*Alloperla petasata* Surdick, 2004. The species occurs in small streams (Fig. 12) in the south-central region of the state (Fig. 25). Adult presence spans mid-May through June (Table 3). Its range traverses the Appalachian Mountains from Georgia northeastward to the Canadian Maritime Provinces and Ontario.

*Alloperla usa* Ricker, 1952. This species resides in three widely separated areas of central and northeastern Ohio (Fig. 25) where it inhabits small streams and rivers (Fig. 12). Adults are available from mid-May through early July (Table 3). This Appalachian Mountain species occurs from Alabama northeastward to Pennsylvania.

*Haploperla brevis* (Banks, 1895). This common species inhabits mainly small streams (Fig. 12) in the eastern half of the state (Fig. 25). Adult presences spans May through mid-August (Table 3). The range of this species encompasses all of eastern North America, extending northwestward to British Columbia.

**Figure 13.**
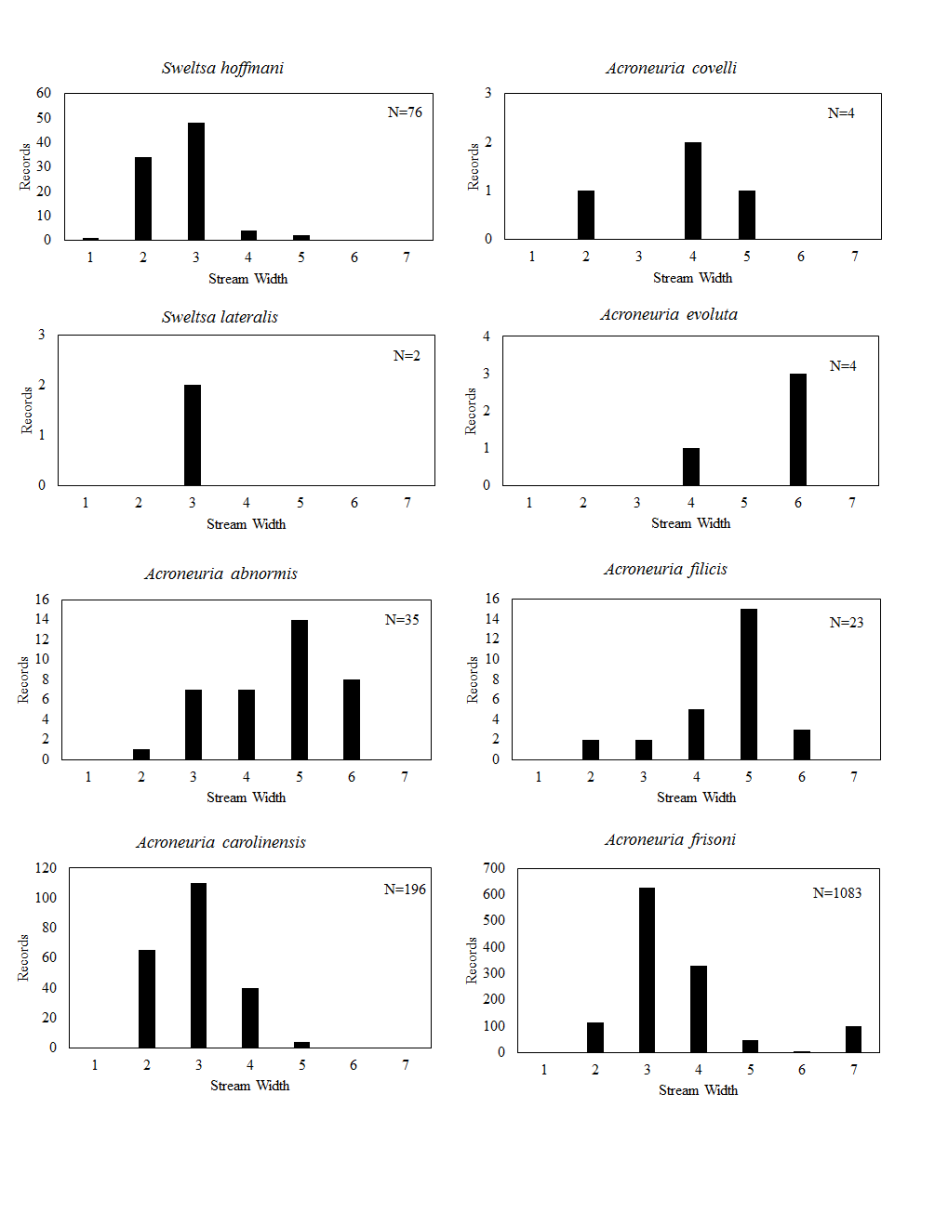
Stream width categories occupied by Chloroperlidae and Perlidae species in Ohio. Stream width categories: 1=seep, 2=1-2 m, 3=3-10 m, 4=11=30 m, 5=31-60 m, 6=>60 m, 7=Lake Erie.

*Sweltsa hoffmani* Kondratieff & Kirchner, 2009. Our analysis demonstrates that this common species most often inhabits small, cool, ravine streams, though some have been reported from medium to large rivers (Fig. 13). The latter is probably an artifact of the use of light traps to collect specimens. The species ranges throughout southern, central, and northeastern Ohio (Fig. 26). Adults occur from mid-March through early July (Table 3). *Sweltsa hoffmani* appears distributed in the western lower elevation plateaus of the Appalachian Mountains, further westward to Indiana, and Kentucky and south to Alabama.

*Sweltsa lateralis* (Banks, 1911). This is another rare species in Ohio. It occurs in small streams (Fig. 13) in southern and eastern Ohio (Fig. 26) with adults appearing in mid-May (Table 3). It is a widespread Appalachian species distributed from Georgia to northeastern Canada.

### Perlidae. Summer Stoneflies

*Acroneuria abnormis* (Newman, 1838). This species uses a wide range of stream sizes with the greatest frequency of records coming from streams 31-60 m wide (Fig. 13). It is mainly distributed east of a line from the southwest to the northeast, but records exist from the far northwest corner of the state in Fish Creek (Williams County) (Fig. 26). Adults are available from June through August (Table 3). This species may have lost range in Ohio, though there are 12 unique locations, mainly from the OEPA, reported since 1990. *Acroneuria abnormis* is widely distributed across North America, being absent only from the warmest, driest, and coldest regions of the West. Larvae of this species are easily confused with that of *A. internata*. Rearing of larvae to adulthood is the best way to confirm identifications.

*Acroneuria carolinensis* (Banks, 1905). This common species generally inhabits smaller streams than *A. abnormis* (Fig. 13), though it occurs over much the same area (Fig. 26). Adult presence spans May through June (Table 3). *Acroneuria carolinensis* is mainly an Appalachian-distributed species known from Mississippi northeast to Quebec and west to eastern Manitoba. Larvae of this species may be confused with *A. lycorias* since both display banding on the posterior half of each abdominal segment. The absence of anal gills confirms the identity of *A. carolinensis*

*Acroneuria covelli* Grubbs & Stark, 2004. This species is rare in Ohio, being known from only three locations in Athens County (Fig. 26). All records date prior to 1942 and specimens probably originated from the Hocking River (Fig. 13). Adult records are for July (Table 3). Although *A. covelli* is considered extirpated from the state, this species is may still be present in the largest rivers in the southern half of the state (DeWalt et al. 2012, Grubbs et al. 2013b). This species inhabits a narrow range that includes Indiana, Kentucky, Ohio, and Tennessee. The larva of this species in unknown.

*Acroneuria evoluta* Klapálek, 1909. Only three adult records of this species exist for Ohio, one from a non-specific location in Adams County, another from Black Lick Creek in Franklin County, and another location, "Catonbads", that cannot be placed (Needham and Claassen 1925), all collected prior to 1937. More recently, Beckett (1987) reported four larvae, collected in 1979, from artificial substrates at the Ohio River Launch Club in Hamilton County (Fig. 26). We accept his identification with some uncertainty because we have not examined the specimens. All specimens came from larger rivers, mostly from the very largest (Fig. 13). Although DeWalt et al. (2012) and Grubbs et al. (2013b) considered the species extirpated from the state, it is possible that the Ohio River may support a few populations. We report adults for June and October (Table 3). *Acroneuria evoluta* occurs broadly across eastern North America, mainly in unglaciated landscapes.

*Acroneuria filicis* Frison, 1942. This species once occurred in a wide variety of stream sizes (Fig. 13), primarily across southern Ohio (Fig. 26). It has experienced a prominent range reduction since the 1950’s, similar to that reported for Illinois (DeWalt et al. 2005). Adults are present during June and July (Table 3). Five records exist post-1977: two from the Grand River in Lake County and one each from the West Fork Straight Creek in Brown County, Crane Hollow Nature Preserve in Hocking County, and Ohio Brush Creek in Adams County. We know this species to be a complex based on a diversity of egg types. The entire complex inhabits a band of unglaciated landscapes from the Interior Highlands eastward to Virginia and south to Alabama and Georgia.

*Acroneuria frisoni* Stark & Brown 1991. This species occurs widely across Ohio (Fig. 26) where it inhabits small streams to medium sized rivers (Fig. 13). Adult presence encompasses May through early July (Table 3). Samples from the OEPA and OBS demonstrate that it is the most common *Acroneuria* in Ohio and that its status is secure. Unfortunately, it has been lost from the wave-swept shores of the Bass Islands in the Western Basin of Lake Erie where it used to be abundant prior to 1950 (Clark 1934, DeWalt et al. 2012, Grubbs et al. 2013b). The species displays an hourglass shaped distribution from the Interior Highlands eastward across a narrow section of suitable habitat in southern Illinois to the western foothills of the Appalachian Mountains, then northward to the glaciated Great Lakes area (Pessino et al. 2014).

**Figure 14.**
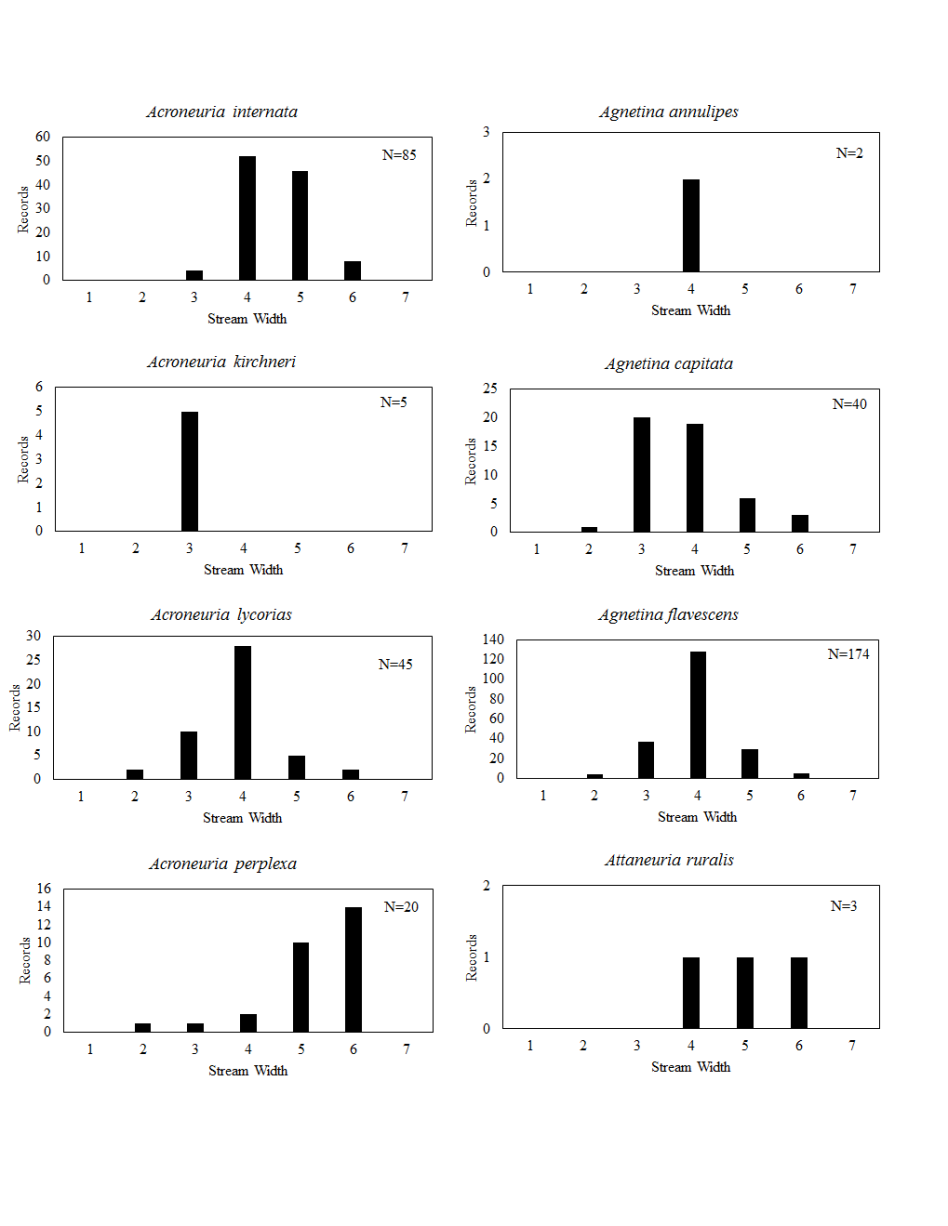
Stream width categories occupied by Perlidae species in Ohio. Stream width categories: 1=seep, 2=1-2 m, 3=3-10 m, 4=11=30 m, 5=31-60 m, 6=>60 m, 7=Lake Erie.

*Acroneuria internata* (Walker, 1852). This species inhabits small and medium sized rivers (Fig. 14) along a line from the southwest to northeast corners of the state (Fig. 27). Adults occur during May and June (Table 3). Its range encompasses Oklahoma and Arkansas north to Minnesota and east to Virginia. Those who work with larvae of this species should be aware that it is easily confused with *A. abnormis* larvae. Rearing is the best way to confirm identifications.

Acroneuria kirchneri Stark & Kondratieff, 2004. This rare species presumably inhabits only small streams (Fig. 14) in the south-central and northeastern regions of the state (Fig. 27). Adult females were found in mid-June through early July (Table 3). Larvae of this species are unknown. Published records of *A. kirchneri* now include Ohio, Kentucky, Pennsylvania, Virginia, and West Virginiafrom KY, PA, VA, WV.

*Acroneuria lycorias*. This species utilizes a wide range of stream sizes (Fig. 14) mainly in the south-central and northeastern regions of the state (Fig. 27). Adult presence is based on only two unique records, both from early July (Table 3) The range of *A. lycorias* extends across most of eastern North America. Larvae of this species are easily confused with *A. carolinensis* since both display banding on the posterior half of each abdominal segment. The presence of anal gills confirms *A. lycorias*.

*Acroneuria perplexa* Frison, 1937. This species is considered extirpated from Ohio since all records span the years 1899 to 1948 (Grubbs et al. 2013b). The species was most frequently collected from large rivers (Fig. 14), mainly in the southern half of the state (Fig. 27). Adults were collected from May through mid-July, but were most abundant in June (Table 3). The range of this species is mostly within large rivers in the Mississippi River drainage from Oklahoma and Georgia into Missouri and eastward to Pennsylvania.

*Agnetina annulipes*. Data for this species are scanty with only two of four records capable of being georeferenced. These two records place it in the Little Miami River near Clifton Falls, a medium sized river in that location (Fig. 14). This location and another in Scioto County suggest that the species colonized the central and southwestern parts of the state (Fig. 27). Records date from 1899 to 1930, so it too is considered extirpated from Ohio (Grubbs et al. 2013b). Adult records are from June and early July (Table 3). This is a Gulf and Atlantic Coastal Plain species that extends northward to Indiana, Ohio, and Pennsylvania.

*Agnetina capitata* (Pictet, 1841). This common species utilizes a wide range of stream sizes (Fig. 14) across most of the state except for the depauperate northwestern counties (Fig. 27). Adult presence spans May through July (Table 3). Its range covers the majority of eastern North America.

*Agnetina flavescens* (Walsh, 1862). This *Agnetina* is also common, occupying similar stream sizes (Fig. 14) and a nearly identical distribution (Fig. 27) to that of *A. capitata*. Adults occur from May through August (Table 3). This species is largely sympatric with *A. capitata*, although its distribution extends slightly further west and south.

*Attaneuria ruralis* (Hagen, 1861). The four Ohio records for this species predate 1926, because of this we consider it extirpated from the state (Grubbs et al. 2013b). All records are from larger rivers (Fig. 14) and adult presence spans June to early July (Table 3). Its distribution encompasses three localities in central and southwestern Ohio (Fig. 27). The overall distribution of this species encompasses large, summer-warm rivers of the Mississippi River drainage and large rivers in the Gulf and Atlantic Coastal Plain.

**Figure 15.**
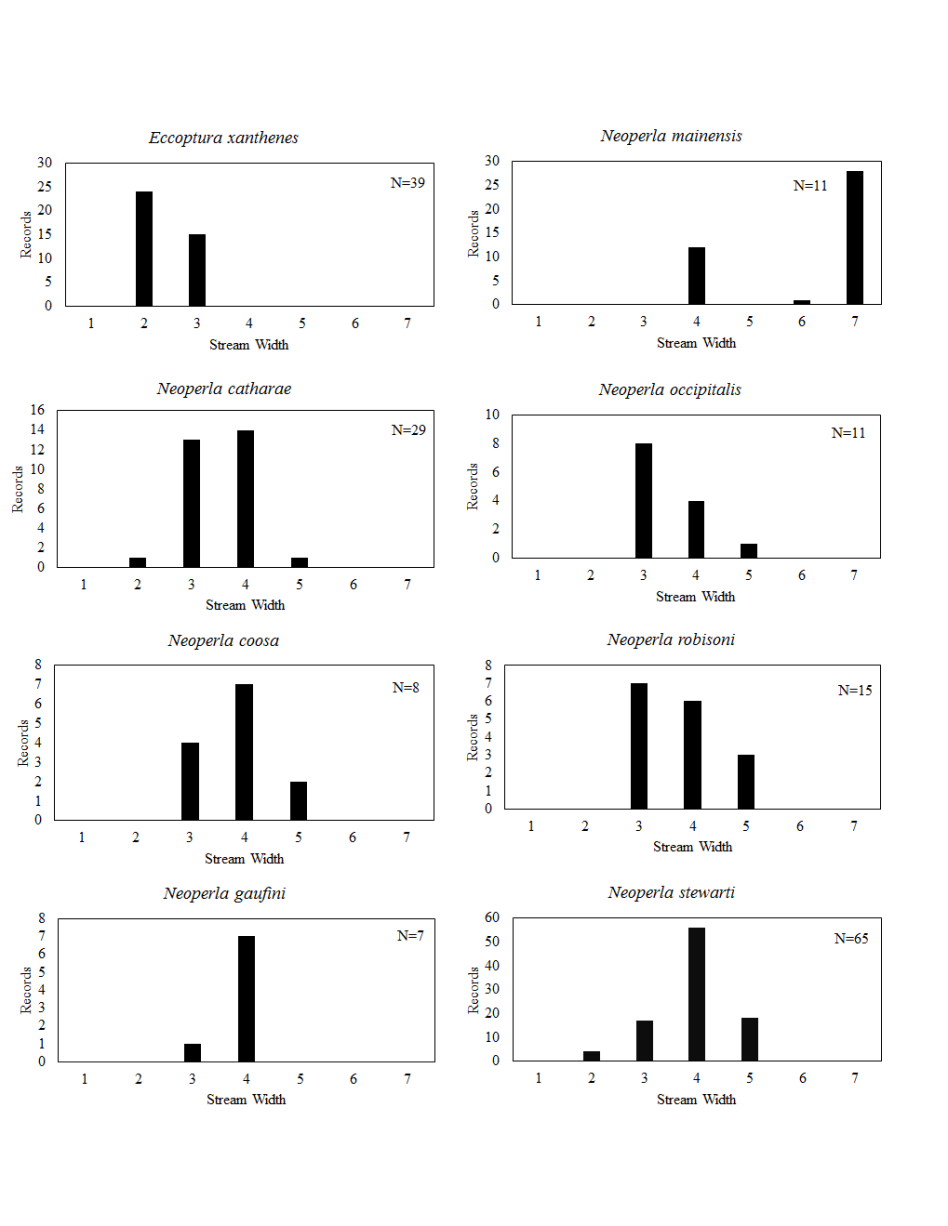
Stream width categories occupied by Perlidae species in Ohio. Stream width categories: 1=seep, 2=1-2 m, 3=3-10 m, 4=11=30 m, 5=31-60 m, 6=>60 m, 7=Lake Erie.

*Eccoptura xanthenes* (Newman, 1838). This species inhabits small, usually ravine associated streams (Fig. 15) in southern and eastern Ohio (Fig. 28). Adults are present during June and July (Table 3). This mainly Appalachian-distributed species occurs from Florida north to New York.

*Neoperla catharae* Stark & Baumann, 1978. This species occurs mainly in medium sized streams and rivers (Fig. 15). Its distribution encompasses the unglaciated southern half of the state with a few records venturing into the glaciated northeast (Fig. 28). This is a late emerging perlid of July and August with adults occurring as late as mid-September (Table 3). The distribution of this species encompasses mainly unglaciated landscapes from the Interior Highlands, eastward to Virginia and Pennsylvania.

*Neoperla coosa* Smith & Stark, 1998. Small streams to medium rivers support this species (Fig. 15) in the southwestern and northeastern regions of the state (Fig. 28). Adult presence spans May to July (Table 3). The distribution of *N. coosa* is widely scattered, and includes Alabama, Indiana, Ohio, New York, North Carolina, and Tennessee. We expect that the scattered pattern is an artifact of its recent description, since adults are easily confused with *N. clymene*.

*Neoperla gaufini* Stark & Baumann, 1978. This is a rare find in Ohio since only four unique locations, all in the southwestern region of the state, are known (Fig. 28). It lives in small streams to small rivers (Fig. 15). Adults occur mostly from June to early July (Table 3). This largely Ohio River Valley species is known only from Indiana, Kentucky, and Ohio.

*Neoperla mainensis* Banks, 1948. Records exist for the Bass Islands of Lake Erie, the Olentangy River near Columbus, and the Clear Fork of the Mohican River near Loudonville (Figs 15, 28). Adult presence spans May through early July (Table 3). Our records range from 1899 to 1922, suggesting that the species has been extirpated from the state (Grubbs et al. 2013b), and possibly the entire region (DeWalt et al. 2005). *Neoperla mainensis* is also known from Illinois, Maine, and Ontario.

*Neoperla occipitalis* (Pictet, 1841). This uncommon species occurs in large streams and medium rivers (Fig. 15) in southwestern, central, and northeastern regions of the state (Fig. 28) Adults are present in June and July (Table 3). This species spans much of eastern North America.

*Neoperla robisoni* Poulton & Stewart, 1986. This species inhabits large streams and medium rivers (Fig. 15), mainly in the southwestern region of the state (Fig. 28). Adults occur from May through August (Table 3). The distribution of this species centers in the Interior Highlands with extensions into the Gulf South, the unglaciated Midwestern states, and eastward to West Virginia and Pennsylvania.

*Neoperla stewarti* Stark & Baumann, 1978. This common species occupies small streams to medium rivers (Fig. 15) with most localities concentrated in southern and central Ohio (Fig. 28). Additionally, many large populations exist in the northeastern direct tributaries of Lake Erie. Adult presence spans May through August (Table 3). This species occurs across much of eastern North America, but has not been reported from Georgia, Florida, or any Canadian province.

**Figure 16.**
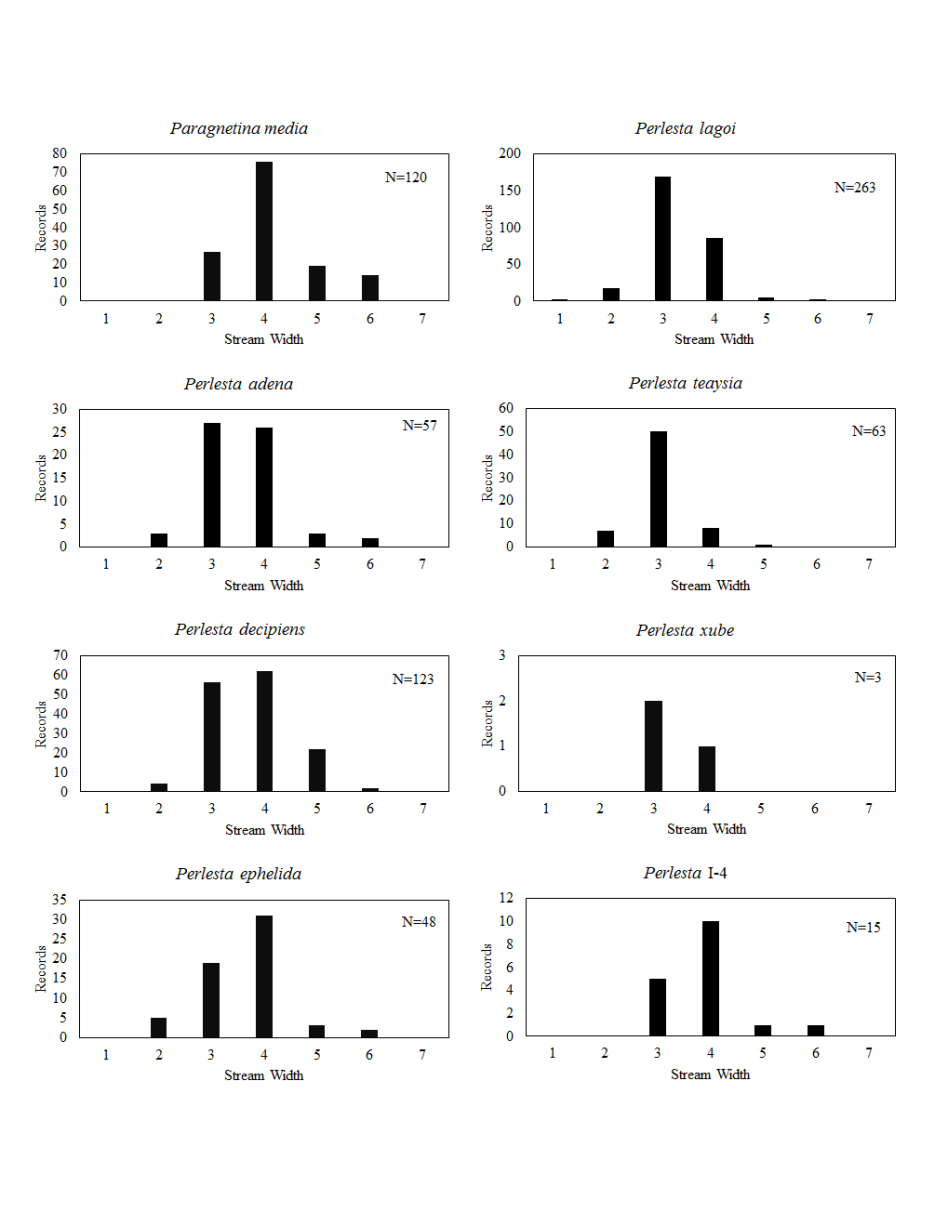
Stream width categories occupied by Perlidae species in Ohio. Stream width categories: 1=seep, 2=1-2 m, 3=3-10 m, 4=11=30 m, 5=31-60 m, 6=>60 m, 7=Lake Erie.

*Paragnetina media* (Walker, 1852). This a common species in Ohio. It inhabits a wide range of stream sizes (Fig. 16) mainly across central and northern regions (Fig. 29). Only three of 137 records were of adults, and of these, all occurred in the second half of May (Table 3). *Paragnetina media* occurs over much of eastern North America and westward into Manitoba and Saskatchewan.

*Perlesta adena* Stark, 1989. This common species inhabits a wide range of stream sizes (Fig. 16). It occurs in all areas of the state except the southeastern quarter (Fig. 29). Although our current data exclude it from this region, there is no reason to doubt its presence in the southeast. Adults occur from mid-May through July (Table 3). *Perlesta adena* ranges mainly in the Ohio River Valley, from Ohio and Indiana south to Tennessee.

*Perlesta decipiens* (Walsh, 1862). This is also a common species and exhibits nearly the same stream size usage (Fig. 16) and geographic distribution as *P. adena* (Fig. 29). Adult presence spans May through August (Table 3). *Perlesta decipiens* is one of the most widely distributed stoneflies in North America.

*Perlesta ephelida* Grubbs & DeWalt, 2012. This species inhabits a large range of stream sizes (Fig. 16) and demonstrates three main clusters of distribution in Ohio: northeast, central, and northwest (Fig. 29). Prior to its description by Grubbs and DeWalt (2012) this species was confused with *P. shubuta* Stark, 1989. Adults occur mainly in June and July (Table 3). *Perlesta ephelida* is distributed across east-central North America from Arkansas to Minnesota, east to Maryland and Massachusetts, and north to Ontario (Grubbs and DeWalt 2012).

*Perlesta lagoi* Stark, 1989. The distribution of this species is statewide (Fig. 29), utilizing small streams to medium rivers (Fig. 16). Adults occur from mid-May through late August (Table 3). The distribution of this species as understood currently is tightly tied to the Mississippi and Ohio river valleys.

*Perlesta teaysia* Kirchner & Kondratieff, 1997. This species utilizes mainly small streams to small rivers in Ohio (Fig. 16) and is often the only *Perlesta* to inhabit small, ravine streams. The species occurs in all parts of the state with the exception of the depauperate northwestern counties (Fig. 29). Adults occur from June through mid-August (Table 3). To date, *P. teaysia* occurs only in a narrow band from Illinois, southward to Tennessee and eastward to Pennsylvania and Virginia.

*Perlesta xube* Stark & Rhodes, 1997. This rare species utilizes large streams to small rivers (Fig. 16). We report it from five central and southwestern border sites in the state (Fig. 29). Adults are present June through mid-July (Table 3). It appears that *P. xube* originated in western Prairie regions and spread eastward to Ohio.

*Perlesta* I-4. This undescribed species inhabits large streams and small rivers (Fig. 16), mainly in western and southern Ohio (Fig. 29). Adults occur in late May through July (Table 3). DeWalt and Grubbs (2011) reported this species in Indiana as *P. cinctipes*.

**Figure 17.**
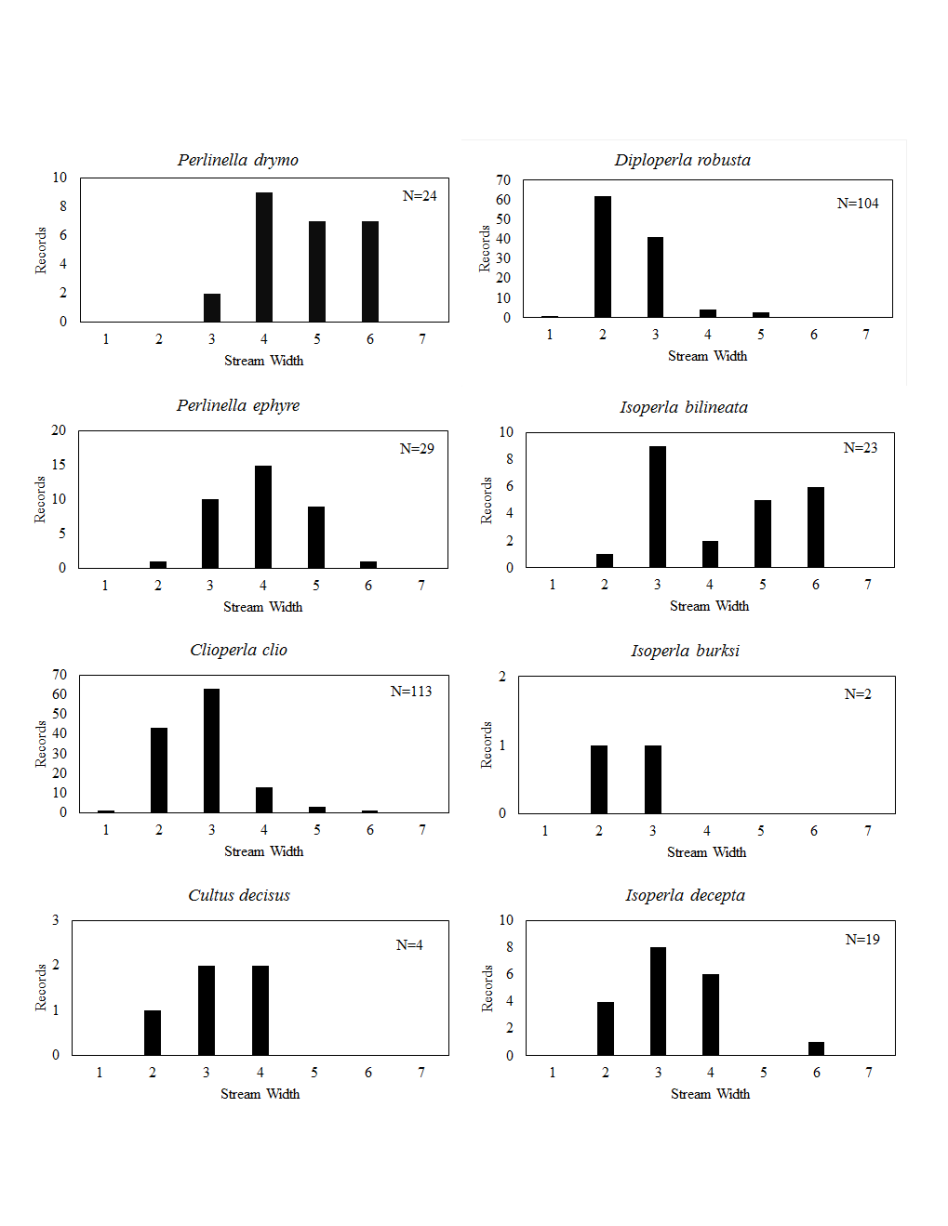
Stream width categories occupied by Perlidae and Perlodidae species in Ohio. Stream width categories: 1=seep, 2=1-2 m, 3=3-10 m, 4=11=30 m, 5=31-60 m, 6=>60 m, 7=Lake Erie.

*Perlinella drymo* (Newman, 1839). This species occurs in the largest streams and rivers (Fig. 17). Our data suggest that it is mainly confined to the southern half of the state (Fig. 30). This distribution may be an artifact of *P. drymo* being one of the earliest-emerging perlids—in a lull of emergence when little collecting takes place. Future efforts, focused in April through early May (Table 3) will undoubtedly increase the known range of this species in Ohio. It ranges from Texas to the Interior Highlands, then eastward to the Atlantic Coast and northward to Minnesota, Quebec and Nova Scotia

*Perlinella ephyre* (Newman, 1839). Large streams and rivers support this species (Fig. 17) in the southern, north-central, and northeastern regions of the state (Fig. 30). Adults are present from May through mid-July, with June producing the majority of specimens (Table 3). This species occupies a similar range to that of *P. drymo*, except that it is apparently absent from Canada.

### Perlodidae. Spring Stones

*Clioperla clio* (Newman, 1839). This common species most often inhabits small to medium sized streams (Fig. 17) nearly everywhere in the state (Fig. 30). Adult presence spans April through June (Table 3). The species ranges from Florida to Texas and northward to Ontario.

*Cultus decisus* (Walker, 1852). This species inhabits four small streams (Fig. 17) in Lake and Geauga counties of northeastern Ohio (Fig. 30). Adults probably occur in May, although there is only one adult record available (Table 3). Given the uncertainty of the species or subspecies represented by these records, a more general distribution for eastern Cultus is presented. *Cultus verticalis* occurs from Tennessee and North Carolina to the Virginias north to New England and Quebec. *Cultus d. isolatus* is a southern Appalachian Mountains species, being known from Georgia, North Carolina, and Virginia. *Cultus d. decisus* is the northern subspecies, being known from Michigan, New York, Pennsylvania, West Virginia, New Brunswick, and Ontario.

*Diploperla robusta* Stark & Gaufin, 1974. This is a small stream species (Fig. 17) distributed widely in southern and eastern Ohio (Fig. 30). Adults are present from mid-April through early June (Table 3). *Diploperla robusta* occurs as far west as eastern Illinois, south to Alabama and northeastward to Virginia and Connecticut.

*Isoperla bilineata* (Say, 1823). This species occurs mainly in larger streams and rivers (Fig. 17) at scattered locations across much of the state (Fig. 30). Adult presence begins in late March, extending into early June (Table 3). The confirmed range of *I. bilineata* includes Saskatchewan and Manitoba in Canada and Iowa, Illinois, Indiana, Kansaw, Michigan, Minnesota, Missouri, Mississippi, North Dakota, Ohio, and Wisconsin in the USA (Szczytko and Kondratieff 2015).

*Isoperla burksi* Frison, 1942. Larvae of this rare Ohio species occur in small streams (Fig. 17) in the southern half of the state (Fig. 30). Adults are available in late May or early June (Table 3). This species is restricted to unglaciated landscapes in Alabama, the Interior Highlands, eastward into the Ohio River Valley of Illinois, Indiana, Ohio, and further east to the Carolinas, the Virginias and Maryland.

*Isoperla decepta* Frison, 1935. This species occurs mainly in small to mid-order streams (Fig. 17) in the central and southwestern regions of the state (Fig. 30). Adults occur in May and June (Table 3). This species occurs from Alabama, into the Interior Highlands and northward into southern Ontario.

**Figure 18.**
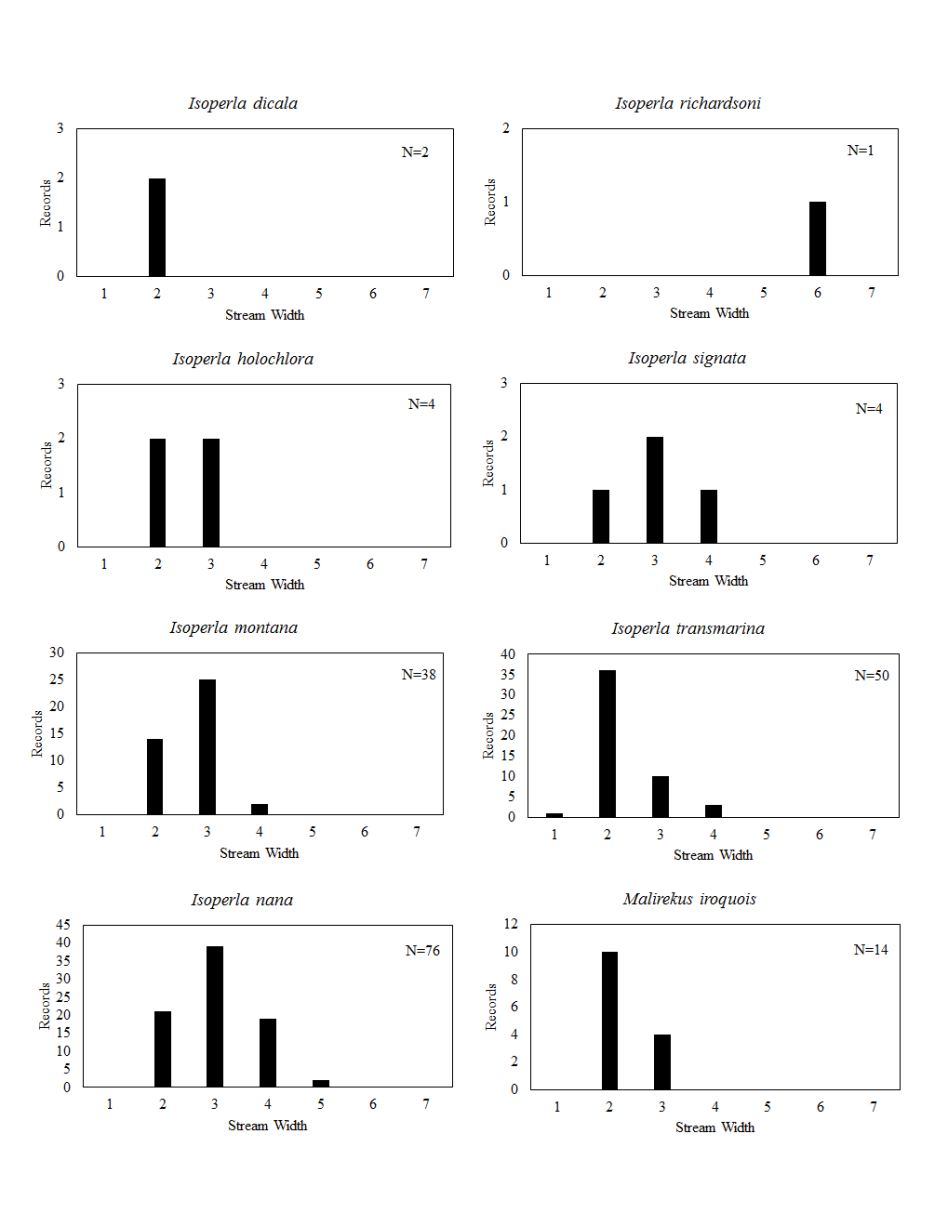
Stream width categories occupied by Perlodidae species in Ohio. Stream width categories: 1=seep, 2=1-2 m, 3=3-10 m, 4=11=30 m, 5=31-60 m, 6=>60 m, 7=Lake Erie.

**Figure 19.**
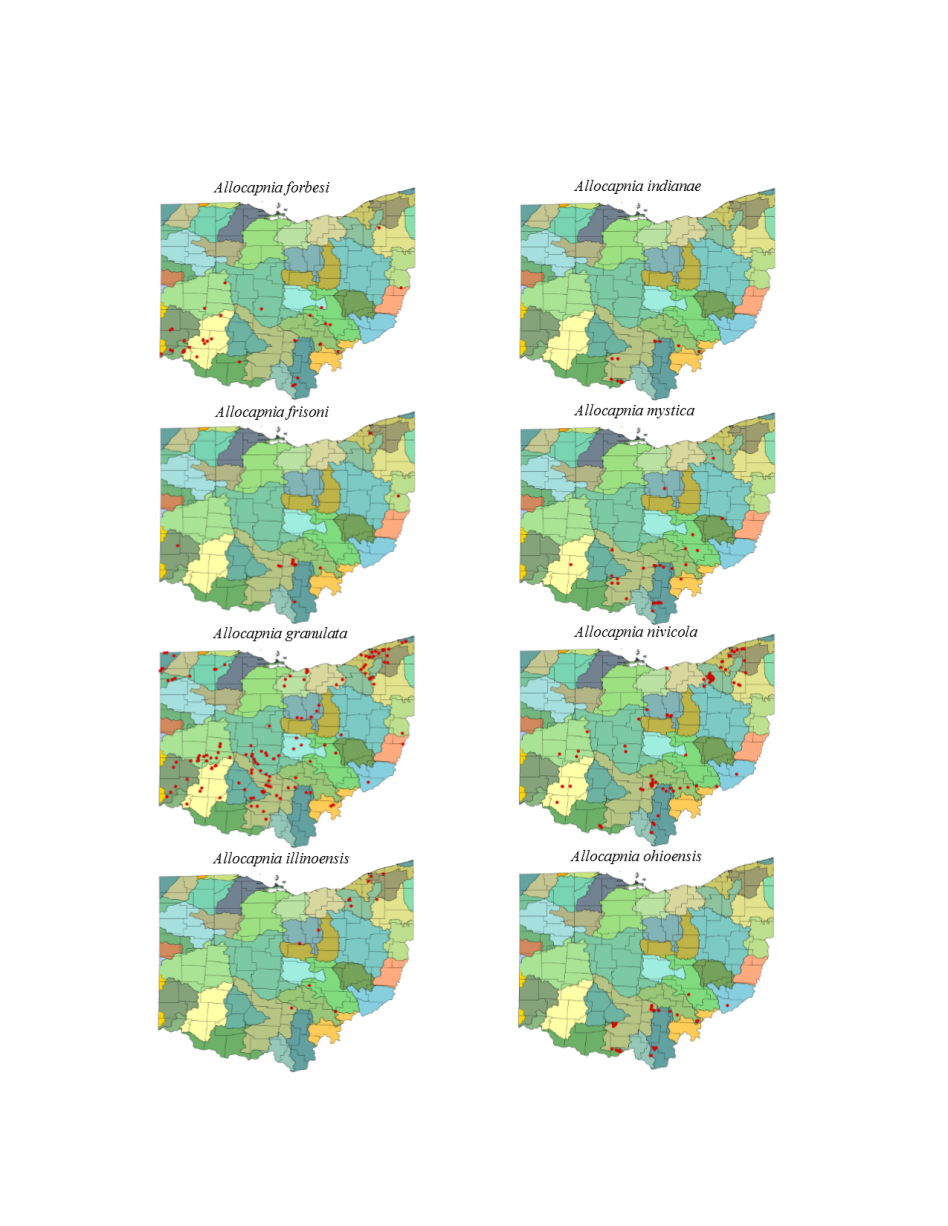
Distribution of Capniidae in Ohio.

**Figure 20.**
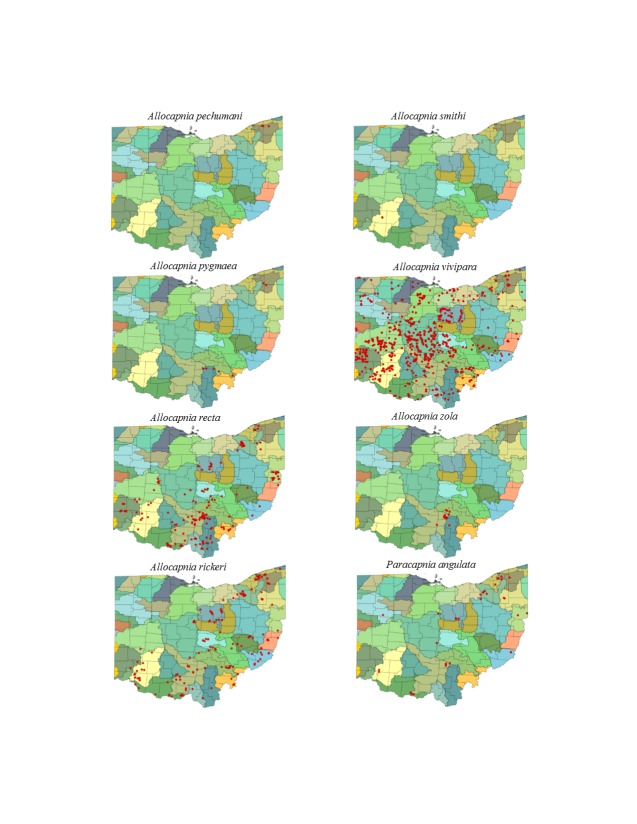
Distribution of Capniidae in Ohio.

**Figure 21.**
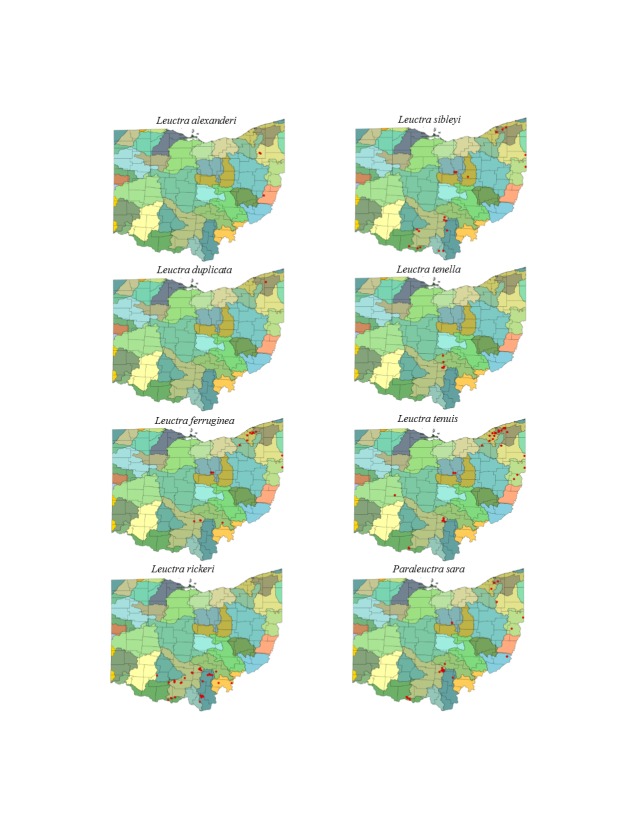
Distribution of Leuctridae in Ohio.

**Figure 22.**
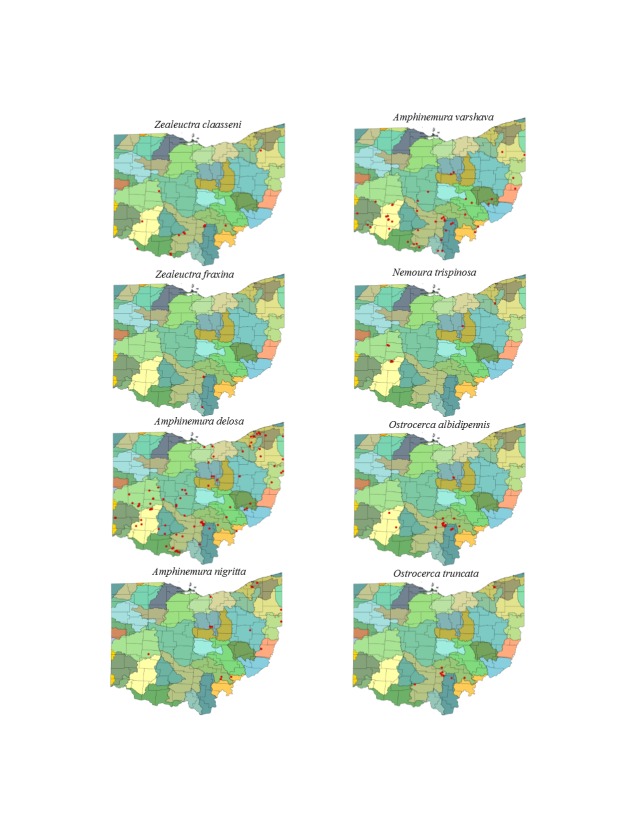
Distribution of Leuctridae and Nemouridae in Ohio.

**Figure 23.**
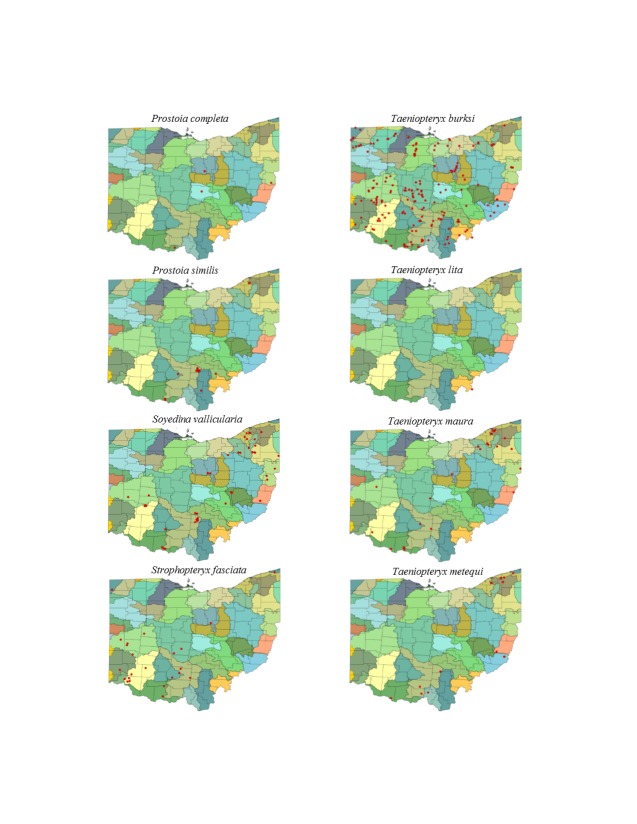
Distribution of Nemouridae and Taeniopterygidae in Ohio.

**Figure 24.**
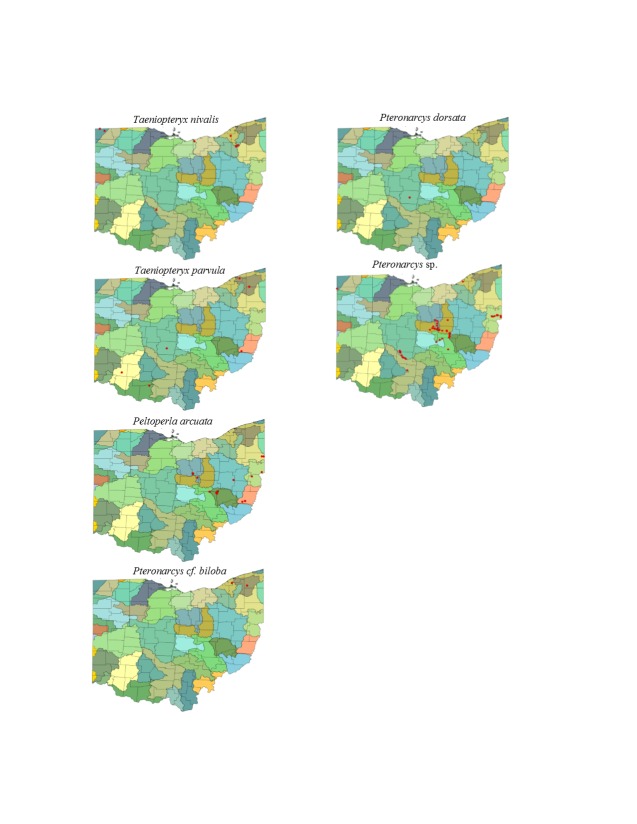
Distribution of Taeniopterygidae, Peltoperlidae, and Pteronarcyidae in Ohio.

**Figure 25.**
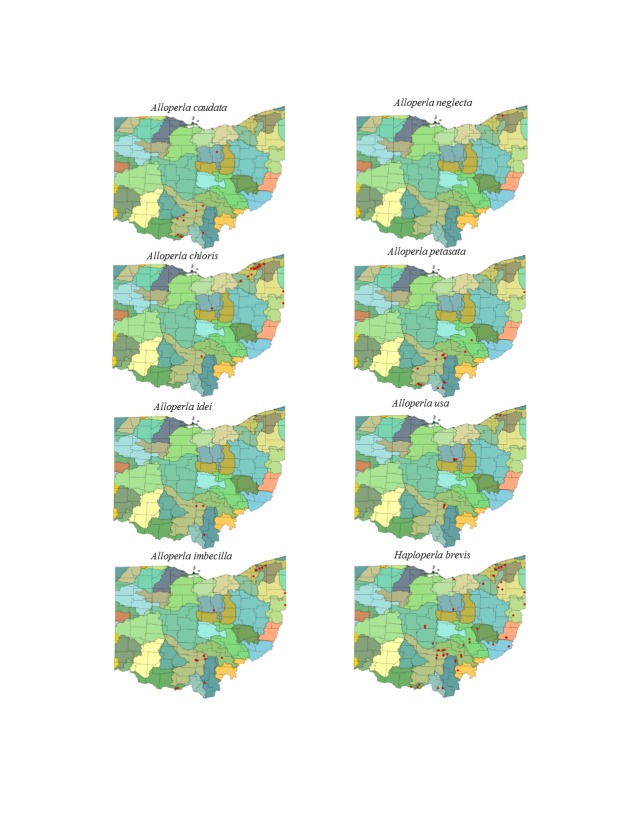
Distribution of Chloroperlidae in Ohio.

**Figure 26.**
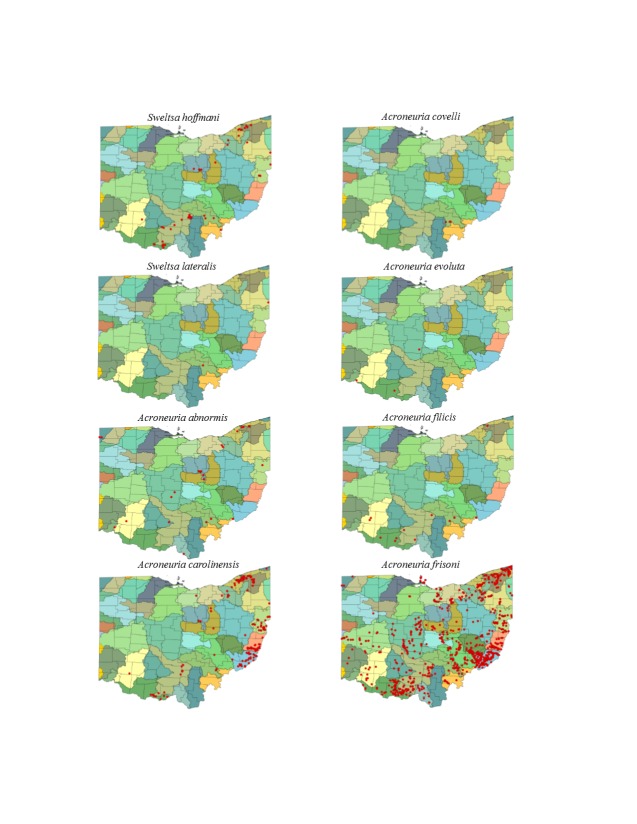
Distribution of Chloroperlidae and Perlidae in Ohio.

**Figure 27.**
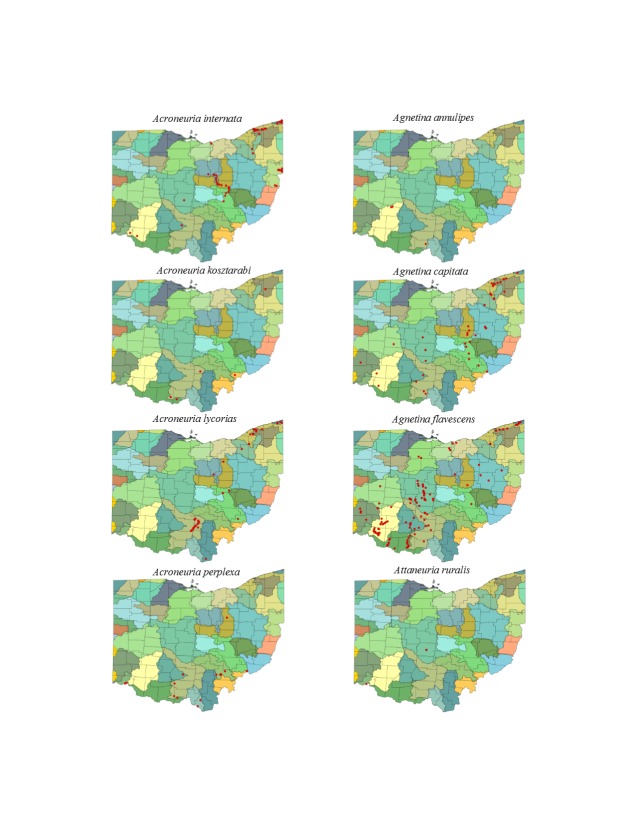
Distribution of Perlidae in Ohio.

**Figure 28.**
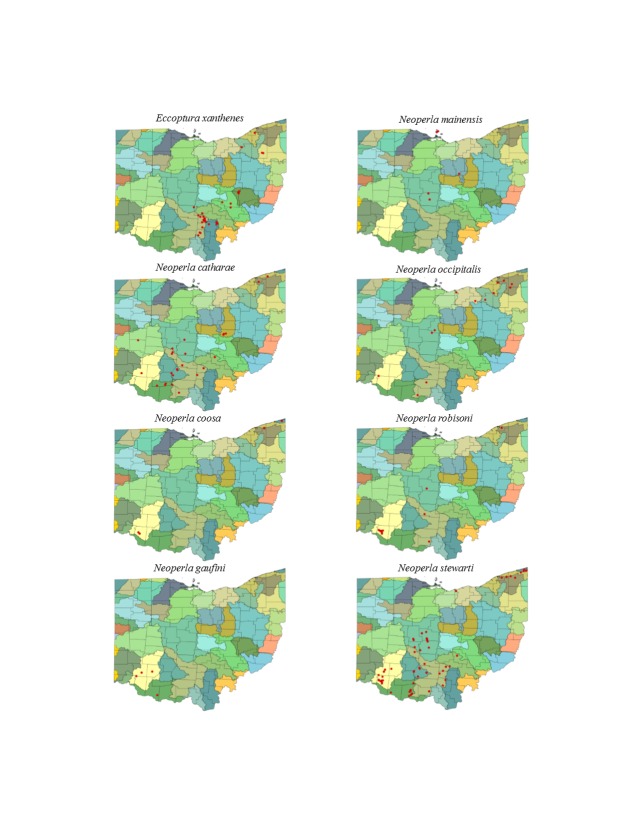
Distribution of Perlidae in Ohio.

**Figure 29.**
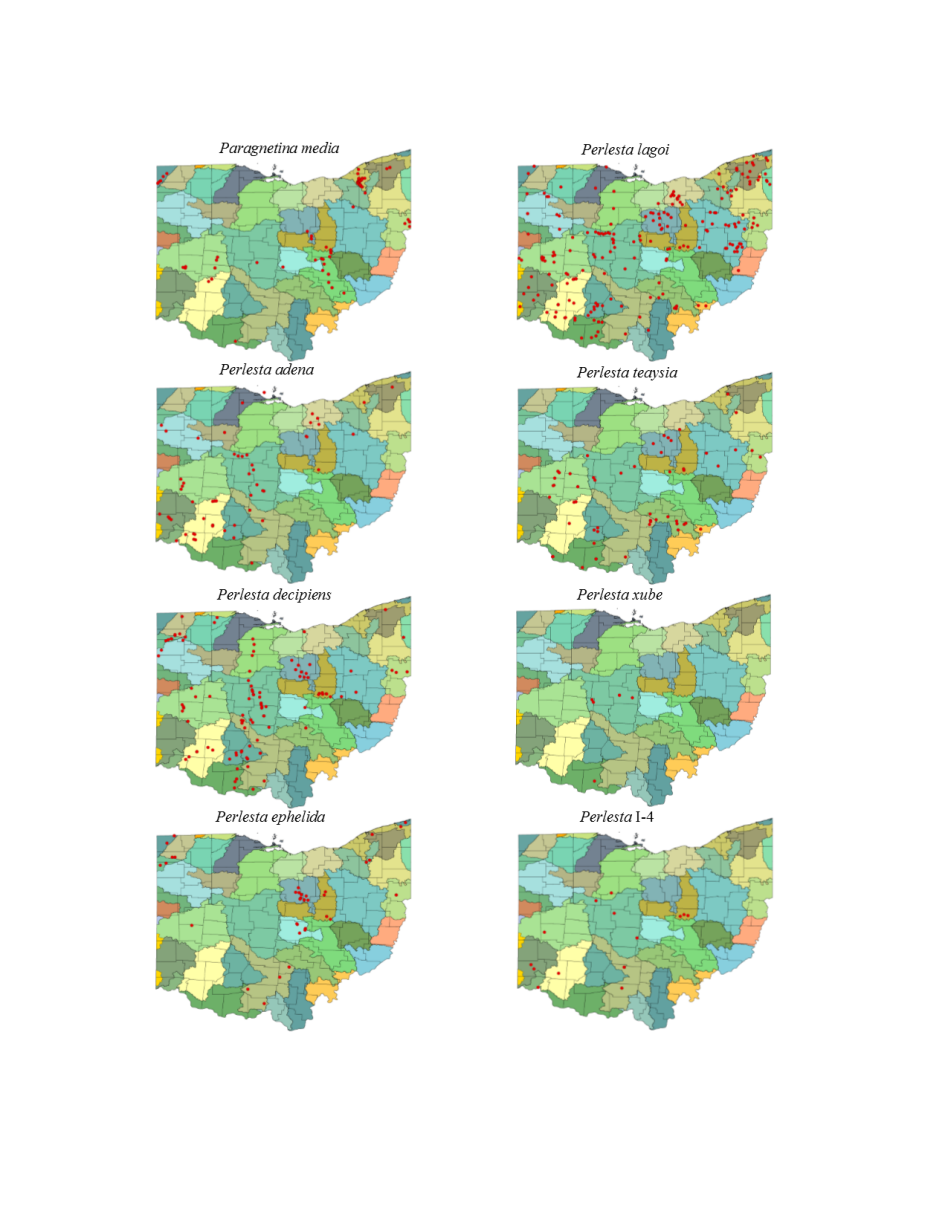
Distribution of Perlidae in Ohio.

**Figure 30.**
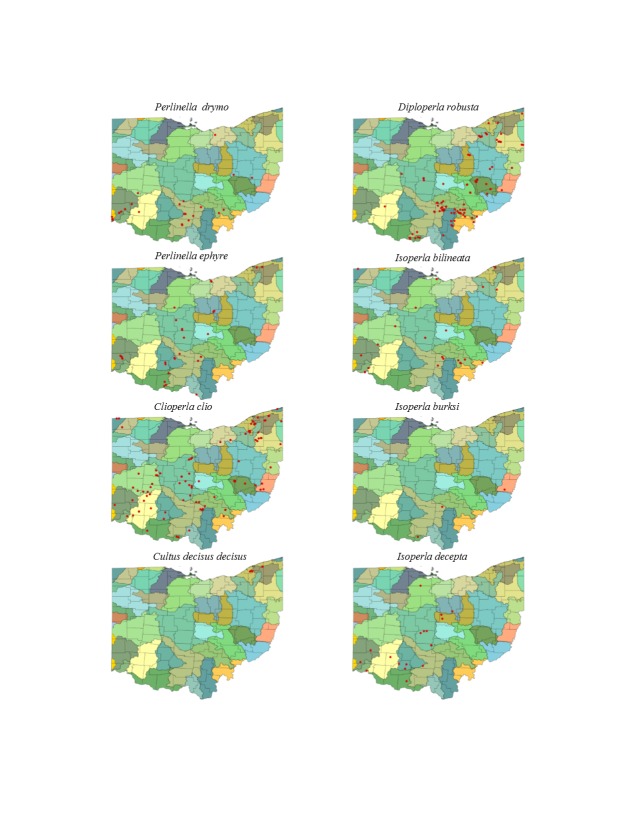
Distribution of Perlidae and Perlodidae in Ohio.

**Figure 31.**
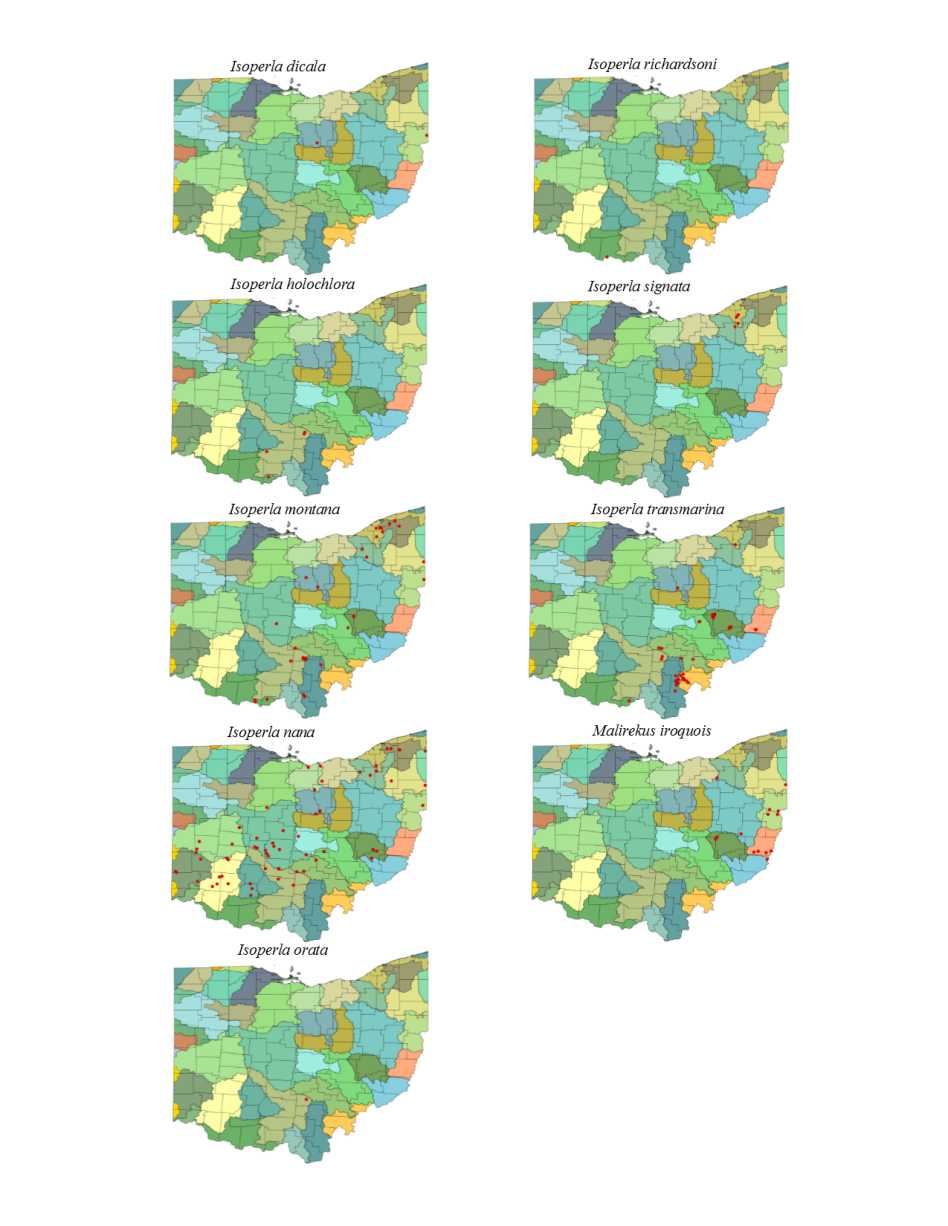
Distribution of Perlodidae in Ohio.

*Isoperla dicala* Frison, 1942. This species is rare in Ohio (Fig. 31) where it inhabits only two small streams (Fig. 18). We know of only one spent adult female from early July, but predict that most adults occur in June (Table 3). Its range encompasses all of eastern North America.

*Isoperla holochlora* Klapálek, 1923. This species too is rare, being known from only four small streams (Fig. 18) in the south-central region (Fig. 31). Adults are available in June (Table 3). This Appalachian species occurs from Alabama and Georgia northeastward to Quebec and Nova Scotia.

*Isoperla montana* (Banks, 1898). This common species inhabits mainly small streams (Fig. 18) from the south-central to the northeastern regions of the state (Fig. 31). Adult presence spans May through mid-July (Table 3). This widespread Appalachian species occurs in Alabama northeastward to Nova Scotia and west to Indiana and Minnesota.

*Isoperla nana* (Walsh, 1862). This common species inhabits small streams to medium sized rivers (Fig. 18) across most of Ohio with exception of the northwestern counties (Fig. 31). Adults are available during May and June (Table 3). *Isoperla nana* ranges from Kentucky northward to Wisconsin and east to Quebec.

*Isoperla orata* Frison, 1942. This is the rarest species in Ohio, being known from only one locality in Crane Hollow Preserve in Hocking County (Fig. 31). Unfortunately, this location was not specific enough to assign to a stream, though it is likely to have come from the 2-3 m wide tributary running through Crane Hollow or the slightly larger Pine Creek nearby. The one adult male is known from early June (Table 3). This species is widely distributed in the Appalachian Mountains from Tennessee and South Carolina northeast to the Canadian Maritime Provinces—a dubious outlier record exists for Minnesota, probably confused with *I. cotta* Ricker, 1952.

*Isoperla richardsoni* Frison, 1935. This is a new state record, confirmed from a single adult female from the Ohio River in Adams County (Figs 18, 31). The specimen is from mid-May (Table 3). This species occurs along a narrow latitudinal belt in or near once-glaciated landscapes from Minnesota south to Missouri and east to Connecticut.

*Isoperla signata* (Banks, 1902). This rare species inhabits only four small streams (Fig. 18) in Geauga and Portage counties (Fig. 31). Though no adults are available, we predict that adults will be found in May and June (Table 3). The distribution of *I. signata* encompasses the Interior Highlands, northward to Manitoba and eastward to the Maritimes of Atlantic Canada.

*Isoperla transmarina* (Newman, 1838). This common species inhabits mainly small streams (Fig. 18) in the eastern half of the state (Fig. 31). We predict that adults occur from mid-May through June (Table 3). This species occurs across a broad northern swath of North America from the Canadian Maritime Provinces west to British Columbia, south to the Ohio River Valley and east to North Carolina.

*Malirekus iroquois* Stark & Szczytko, 1988. Larvae inhabit only small streams (Fig. 18), mostly in the eastern third of the state (Fig. 31). In addition, a single adult female was reared on 12 May 2016 from Little Lyons Creek in Ashland County, considerably further west of other locations. We assume that adults begin emergence in May (Table 3). *Malirekus iroquois* inhabits the Appalachian Mountains from Maryland, Pennsylvania, and Ohio, northward to New York, New Hampshire, Vermont, Quebec, and New Brunswick.​

## Discussion

Our present study added 3717 records to the data set of DeWalt et al. (2012). Close examination of the data from the previous paper demonstrated that three perlid species, *A. kosztarabi*, *N. clymene*, and *P. golconda*, should be removed from the state list, while *A. kirchneri*, *I. orata* and *I. richardsoni* were added. Hence, no absolute change in the number of species has occurred so that the number of species remains at 102. Recent study in Edge of Appalachia Preserve and examination of a large collection from Crane Hollow Nature Preserve added records for some rare species such as *P. angulata* and *L. tenella*, and provided the *I. orata* record. The statewide OEPA collecting efforts in all stream sizes added many locations for pteronarcyids, perlids, and perlodids for which few prior records existed. We expect that a few more species will be found in southern and eastern Ohio. In addition, we still desire adults to confirm the presence/identity of the following species: *T. lita*, P. cf. biloba, *P. dorsata* group members, *A. neglecta*, *Perlesta* I-4, and *C. decisus*.

Most portions of the state were satisfactorily sampled (Fig. 1) and the results correlate well with DeWalt et al. (2012). Both works confirmed that the richest areas of the state were in the south-central, southern, and northeastern portions (Fig. 2), whose topography was either unaffected or mildly affected by Quaternary glacial events. The lower Scioto River was the richest drainage (Figs 2, 3, 4, 5). Alternatively, northwestern drainages and counties were still the most depauperate of stoneflies (Figs 2, 5) where glacial impacts were most severe and the post-glacial Black Swamp (Kaatz 1955) was unsuitable habitat for stoneflies. DeWalt et al. (2012) remarked on the paucity of data available for northwestern Ohio, saying that the reduced stonefly richness was likely due to historically poor habitat. Low richness tallies have persisted there despite the statewide sampling scheme of the OEPA. The glacial lake plain habitat with low slope and fine-grained sediments does not support a rich stonefly fauna. However, Fish Creek, in the far northwest corner benefits from higher slope drift plain habitat, coarser sediments, and higher rates of groundwater recharge. These characteristics double its richness from that of adjacent drainages and is consistent with richness in adjacent Indiana drainages (DeWalt and Grubbs 2011).

The use of museum specimens and agency data was exceedingly valuable for this project. Less than 600 records (7.7%) were added as new specimens to this project by RED and SAG since 2005. Existing data were sufficient to characterize the assemblage to a relatively fine scale. This was perhaps an extraordinary situation with coauthors having started this project decades ago (BJA, RWB, SMC) or providing a continuous source of agency data (MJB) with high confidence identifications. Our experience should give others confidence that they too could obtain enough material to characterize a region given the presence of regional museums and trusted agency data.

Little stonefly data were present in GBIF and iDigBio, other than what was already provided by the INHS. Regional collections had not digitized their material in time for our use. We agree that with time and diligent work by plecopterologists, GBIF will become an important source of stonefly data in the future. To this end, we support the mission of GBIF and iDigBio by providing our data in Darwin Core Archive format from the INHS portal and through an archived data set (DeWalt et al. 2016b). We agree that building resources through these data aggregators is an important endeavor (Sikes et al. 2016). Data from global aggregators should be heavily scrutinized for metadata such as who identified the material, when it was identified, and what life stages were available to support a given determination. Many of the specimens we examined had not been viewed for over 50 years. An unknown but substantially large percentage of the specimens were incompletely identified, unidentified, misidentified, or required some upgrade in their nomenclature in order to make the records useful for our purposes. We suggest that data from GBIF and iDigBio be used as a starting point to accumulate data and identify sources of specimens for loan.

Some state water quality agencies support robust biological monitoring programs where well trained aquatic macroinvertebrate taxonomists are employed and where specimens are vouchered to ensure replicability of the science conducted. Unfortunately, there are many governments that either do not have the resources or believe this level of work is unnecessary to achieve their goals. These organizations only meet the objectives of determining attainment of use designations and adherence to permit regulations. Alternatively, because the OEPA hires qualified taxonomists and vouchers specimens, they also meet the additional objectives of providing data for biodiversity analyses and conservation status assessment. The OEPA has earned a lofty reputation because they set the standard for the water quality monitoring community.

## Conclusions

This work culminates at least 91 years (Needham and Claassen 1925 to present) of stonefly research in Ohio. Despite a large human population, industrialized past, and agricultural dominance in some areas, the Ohio stonefly fauna still reflects the historical biogeography of the state. We have built a 7797 record species-level data set the likes of which are not available anywhere for a similarly sized geopolitical unit. Despite an 89% increase in the number of records from the DeWalt et al. (2012) effort, only two additional species were found, though many new locations for rare and uncommon species are now known. We predict that only a few more species will be found in Ohio, and feel that this work is an invaluable baseline for future research on Ohio Plecoptera, especially for conservation status assessment.

## Supplementary Material

Supplementary material 1 Current Ohio stoneflies, microcitations of known works with names used and count of species. Names reconciled in text. Data type: Literature occurrences
Brief description: All known references were tracked beginning in 1925 with the first work to mention stoneflies from Ohio. Names used and reconciliation of names presented. A total of 53 references, denoted as microcitations (full references in text), are presented within this pdf document.
File: oo_108502.pdf R. E. DeWalt, S. A. Grubbs

## Figures and Tables

**Table 1.** Specimen source, institutional coden, the number of specimen records for each source, and the total number of specimens recorded. *OEPA number of specimens is a severe underestimate since most of the data inadvertently lacked numbers of individuals. Numbers of OEPA specimens reflect only those specimens loaned to RED by OEPA.

**Institution**	**Coden**	#**Records**	#**Specimens**
Illinois Natural History Survey, Champaign	INHS	2072	9623
Ohio Environmental Protection Agency, Grove City	OEPA	1744	142*
Bean Museum, Brigham Young University, Provo, Utah	BYUC	1168	18863
Literature		892	6517
Ohio Biological Survey (from DeWalt et al. 2012)	OBS-INHS	573	2690
Ohio State University, Columbus	OSUC	468	668
Crane Hollow Preserve Collection, Athens, Ohio	CHPC	287	830
Western Kentucky University, Bowling Green	WKUC	170	873
Fred Kirchner, Huntington, West Virginia	RFKC	164	857
Cleveland Museum of Natural History, Cleveland, Ohio	CLEV	67	172
Canadian National Collection, Ottawa	CNC	46	252
Ohio Historical Society Collection, Columbus	OHSC	17	17
Field Museum of Natural History, Chicago, Illinois	FMNH	13	40
Michigan State University, East Lansing	MSUC	10	62
Purdue University Ent. Res. Coll., West Lafayette, Indiana	PERC	7	18
Bill P. Stark Collection, Clinton, Mississippi	BPSC	6	81
Iowa State University, Ames	ISUC	4	6
Royal Ontario Museum, Toronto, Canada	ROME	3	15
University of Michigan Museum of Zoology, Ann Arbor	UMMZ	3	3
University of Minnesota, St. Paul	UMSP	6	18
Cincinnati Museum of Natural History, Ohio	CNHM	2	2
Southern Illinois University, Carbondale	SIUC	1	5
	**Total**	7797	41828

**Table 2.** Stonefly names associated with Ohio since Needham and Claassen (1925). See Suppl. material 1 for accounting of all 53 works to ever list stoneflies for Ohio.

**All names associated with Ohio**	**Comment**	**Current**
** Capniidae **		
*Allocapnia forbesi* Frison		1
*Allocapnia frisoni* Ross & Ricker		1
*Allocapnia granulata* Claassen		1
*Allocapnia illinoensis* Frison		1
*Allocapnia indianae* Ricker		1
*Allocapnia mystica* Frison		1
*Allocapnia nivicola* (Fitch)		1
*Allocapnia ohioensis* Ross & Ricker		1
*Allocapnia pechumani* Ross & Ricker		1
*Allocapnia pygmaea* (Burmeister)		1
*Allocapnia recta* (Claassen)		1
*Allocapnia rickeri* Frison		1
*Allocapnia smithi* Ross & Ricker		1
*Allocapnia vivipara* (Claassen)		1
*Allocapnia zola* Ricker		1
*Capnia vernalis* (Newport)	misidentified *P. angulata*?	
*Paracapnia angulata* Hanson		1
** Leuctridae **		
*Leuctra alexanderi* Hanson		1
*Leuctra duplicata* Claassen		1
*Leuctra ferruginea* (Walker)		1
*Leuctra monticola* Hanson	misidentified *L. alexanderi*?	
*Leuctra rickeri* James		1
*Leuctra sibleyi* Claassen		1
*Leuctra tenella* Provancher		1
*Leuctra tenuis* (Pictet)		1
*Paraleuctra sara* (Claassen)		1
*Zealeuctra claasseni* (Frison)		1
*Zealeuctra fraxina* Ricker & Ross		1
** Nemouridae **		
*Amphinemura delosa* (Ricker)		1
*Amphinemura nigritta* (Provancher)		1
*Amphinemura varshava* (Ricker)		1
*Nemoura trispinosa* Claassen		1
*Ostrocerca albidipennis* (Walker)		1
*Ostrocerca truncata* (Claassen)		1
*Prostoia completa* (Walker)		1
*Prostoia similis* (Hagen)		1
*Soyedina vallicularia* (Wu)		1
** Taeniopterygidae **		
*Strophopteryx fasciata* (Burmeister)		1
*Taenionema atlanticum* Ricker & Ross	not present	
*Taeniopteryx burksi* Ricker & Ross		1
*Taeniopteryx lita* Frison		1
*Taeniopteryx maura* (Pictet)		1
*Taeniopteryx metequi* Ricker & Ross		1
*Taeniopteryx nivalis* Fitch		1
*Taeniopteryx parvula* Banks		1
** Peltoperlidae **		
*Peltoperla arcuata* Needham		1
** Pteronarcyidae **		
*Pteronarcys cf. biloba* Newman	nymphs only	1
*Pteronarcys dorsata* (Say)	confirmed	1
*Pteronarcys pictetii* Hagen	not confirmed	
** Chloroperlidae **		
*Alloperla caudata* Frison		1
*Alloperla chloris* Frison		1
*Alloperla idei* Ricker		1
*Alloperla imbecilla* (Say)		1
*Alloperla neglecta* Frison	continued uncertainty	1
*Alloperla petasata* Surdick		1
*Alloperla usa* Ricker		1
*Haploperla brevis* (Banks)		1
*Sweltsa mediana* Banks	misidentified *S. hoffmani*	
*Sweltsa hoffmani* Kondratieff & Kirchner		1
*Sweltsa lateralis* (Banks)		1
*Sweltsa onkos* (Ricker)	misidentified *S. hoffmani*	
** Perlidae **		
*Acroneuria abnormis* (Newman)		1
*Acroneuria carolinensis* (Banks)		1
*Acroneuria covelli* Grubbs & Stark		1
*Acroneuria evoluta* Klapálek		1
*Acroneuria filicis* Frison		1
*Acroneuria frisoni* Stark & Brown		1
*Acroneuria internata* (Walker)		1
*Acroneuria kirchneri* Stark & Kondratieff		1
*Acroneuria kosztarabi* Kondratieff & Kirchner	misidentified *A. kirchneri*	
*Acroneuria lycorias* (Newman)		1
*Acroneuria perplexa* Frison		1
*Agnetina annulipes* (Hagen)		1
*Agnetina capitata* (Pictet)		1
*Agnetina flavescens* (Walsh)		1
*Attaneuria ruralis* (Hagen)		1
*Eccoptura xanthenes* (Newman)		1
*Neoperla catharae* Stark & Baumann		1
*Neoperla clymene* (Newman)	nymphs only, removed from list	
*Neoperla coosa* Stark & Smith		1
*Neoperla gaufini* Stark & Baumann		1
*Neoperla mainensis* Banks		1
*Neoperla occipitalis* (Pictet)		1
*Neoperla robisoni* Poulton & Stewart		1
*Neoperla stewarti* Stark & Baumann		1
*Paragnetina media* (Walker)		1
*Perlesta adena* Stark		1
*Perlesta cinctipes* (Banks)	referable to *Perlesta* I-4	
*Perlesta decipiens* (Walsh)		1
*Perlesta ephelida* Grubbs & DeWalt		1
*Perlesta golconda* DeWalt & Stark	removed from list	
*Perlesta lagoi* Stark	*lagoi* & *nitida* may be a cline	1
*Perlesta nitida* Banks	*lagoi* & *nitida* may be a cline	
*Perlesta placida* (Hagen)	any one of 7 spp. possible	
*Perlesta teaysia* Kirchner & Kondratieff		1
*Perlesta xube* Stark & Rhodes		1
*Perlesta* I–4	new, dark species	1
*Perlinella drymo* (Newman)		1
*Perlinella ephyre* (Newman)		1
** Perlodidae **		
*Clioperla clio* (Newman)		1
*Cultus decisus* (Walker)	uncertain specific/subspecific identity	1
*Diploperla robusta* Stark & Gaufin		1
*Isoperla bilineata* (Say)		1
*Isoperla burksi* Frison		1
*Isoperla decepta* Frison		1
*Isoperla dicala* Frison		1
*Isoperla holochlora* (Klapálek)		1
*Isoperla montana* (Banks)		1
*Isoperla namata* Frison	referable to *I. montana*	
*Isoperla nana* (Walsh)		1
*Isoperla orata* Frison	new state record	1
*Isoperla richardsoni* Frison	new state record	1
*Isoperla signata* (Banks)		1
*Isoperla transmarina* (Newman)		1
*Malirekus iroquois* Stark & Szczytko	identity confirmed from Ashland Co.	1
Total		102

**Table 3.** Succession of adult presence of Ohio stonefly species. Darkest shade of gray indicates weeks with at least one collecting events with ≥ 3 adults. Lighter gray indicates weeks with events containing ≤ 2 adults. Lightest gray is suggestive of when emergence would take place since no adult specimens were obtained. Events = number of site/date collecting events (date+location). Family abbreviations: CA=Capniidae, CH=Chloroperlidae, L=Leuctridae, N=Nemouridae, P=Perlidae, PE=Perlodidae, PL=Peltoperlidae, PT=Pteronarcyidae, T=Taeniopterygidae.

Taxon	Fam.	XI				XII				I				II				III				IV				V				VI				VII				VIII				IX				X				Events
Allocapnia recta	CA																																																	221
Allocapnia nivicola	CA																																																	90
Allocapnia frisoni	CA																																																	17
Allocapnia forbesi	CA																																																	38
Allocapnia rickeri	CA																																																	151
Allocapnia vivipara	CA																																																	566
Allocapnia illinoensis	CA																																																	12
Allocapnia mystica	CA																																																	32
Allocapnia granulata	CA																																																	28
Allocapnia indianae	CA																																																	14
Allocapnia ohioensis	CA																																																	35
Allocapnia smithi	CA																																																	2
Allocapnia pygmaea	CA																																																	8
Allocapnia pechumani	CA																																																	4
Allocapnia zola	CA																																																	19
Paracapnia angulata	CA																																																	41
Taeniopteryx burksi	T																																																	197
Taeniopteryx maura	T																																																	31
Taeniopteryx metequi	T																																																	14
Soyedina vallicularia	N																																																	37
Taeniopteryx nivalis	T																																																	10
Taeniopteryx parvula	T																																																	7
Strophopteryx fasciata	T																																																	15
Taeniopteryx lita	T																																																	1
Prostoia similis	N																																																	19
Prostoia completa	N																																																	9
Zealeuctra fraxina	L																																																	5
Zealeuctra claasseni	L																																																	16
Paraleuctra sara	L																																																	37
Leuctra sibleyi	L																																																	41
Ostrocerca truncata	N																																																	11
Ostrocerca albidipennis	N																																																	19
Nemoura trispinosa	N																																																	14
Amphinemura delosa	N																																																	111
Perlinella drymo	P																																																	4
Sweltsa hoffmani	CH																																																	21
Isoperla bilineata	PE																																																	24
Diploperla robusta	PE																																																	34
Clioperla clio	PE																																																	23
Amphinemura varshava	N																																																	51
Amphinemura nigritta	N																																																	27
Isoperla nana	PE																																																	52
Isoperla signata	PE																																																	0
Malirekus iroquois	PE																																																	1
*Pteronarcys cf. biloba*	PT																																																	0
Pteronarcys dorsata	PT																																																	1
Leuctra tenella	L																																																	7
Isoperla richardsoni	PE																																																	1
Acroneuria evoluta	P																																																	5
Leuctra alexanderi	L																																																	5
Leuctra duplicata	L																																																	2
Cultus decisus	PE																																																	1
Isoperla burksi	PE																																																	1
Isoperla dicala	PE																																																	1
Isoperla holochlora	PE																																																	1
Isoperla orata	PE																																																	1
Sweltsa lateralis	CH																																																	1
Alloperla neglecta	CH																																																	1
Alloperla idei	CH																																																	3
Isoperla transmarina	PE																																																	0
Peltoperla arcuata	PL																																																	6
Paragnetina media	P																																																	3
Isoperla decepta	PE																																																	10
Isoperla montana	PE																																																	19
Alloperla caudata	CH																																																	10
Haploperla brevis	CH																																																	55
Alloperla chloris	CH																																																	26
Acroneuria frisoni	P																																																	140
Acroneuria carolinensis	P																																																	13
Acroneuria filicis	P																																																	35
Neoperla gaufini	P																																																	7
Perlinella ephyre	P																																																	33
Acroneuria perplexa	P																																																	25
Agnetina capitata	P																																																	16
Agnetina flavescens	P																																																	66
Neoperla mainensis	P																																																	8
Neoperla stewarti	P																																																	74
Perlesta decipiens	P																																																	131
Acroneuria internata	P																																																	5
Alloperla imbecilla	CH																																																	25
Alloperla petasata	CH																																																	21
Alloperla usa	CH																																																	13
Attaneuria ruralis	P																																																	3
Leuctra ferruginea	L																																																	34
Leuctra rickeri	L																																																	39
Perlesta adena	P																																																	61
Perlesta lagoi	P																																																	281
Neoperla robisoni	P																																																	16
Perlesta sp. I–4	P																																																	17
Acroneuria abnormis	P																																																	33
Perlesta ephelida	P																																																	53
Perlesta teaysia	P																																																	73
Perlesta xube	P																																																	6
Agnetina annulipes	P																																																	4
Acroneuria covelli	P																																																	3
Acroneuria kosztarabi	P																																																	5
Acroneuria lycorias	P																																																	3
Eccoptura xanthenes	P																																																	11
Neoperla occipitalis	P																																																	13
Neoperla coosa	P																																																	7
Neoperla catharae	P																																																	37
Leuctra tenuis	L																																																	79
