# Supplementary material for: Atlas of Ohio Aquatic Insects: Volume II, Plecoptera
Source: Biodivers Data J. 2016 Nov 16;(4):e10723. doi: 10.3897/BDJ.4.e10723 (PMC5136677; doi:10.3897/BDJ.4.e10723)
Supplement: Supplementary material 1 — Current Ohio stoneflies, microcitations of known works with names used and count of species. Names reconciled in text. [file biodiversity_data_journal-4-e10723-s001.pdf]

Supplementary Doc1. Current Ohio stoneflies, microcitations of known works with names used and count of species. Names reconciled in text.

Ohio Stoneflies	Current	Need. & Claa.1925	Clark 1935	Frison 1942	Walker 1947	Ricker 1952	Gaufin 1956	Ross & Ricker 1964	Ross & Yama. 1967	Ricker & Ross 1968	Ricker & Ross 1969	Ross & Ricker 1971	Zwick 1971	Baumann 1974	Stark & Gaufin 1974	Stark & Gaufin 1976	Stark & Baum. 1978	Szc. & Stew. 1978	Tkac & Foote 1978	Robertson 1979	Tkac 1979	Full. & Stew. 1980	Szc. & Stew. 1981	Robertson 1984	Stark 1986	Beckett 1987	Fishbeck 1987
**Capniidae**																											
Allocapnia forbesi	1				1		1					1															
Allocapnia frisoni	1											1							1		1						1
Allocapnia granulata	1				1		1		1			1							1		1						1
Allocapnia illinoensis	1											1							1		1						1
Allocapnia indianae	1					1	1					1															
Allocapnia mystica	1											1															
Allocapnia nivicola	1						1					1							1		1						
Allocapnia ohioensis	1							1				1															
Allocapnia pechumani	1																				1						
Allocapnia pygmaea	1			1	1		1														1						
Allocapnia recta	1						1					1							1		1						1
Allocapnia rickeri	1			1	1		1					1							1		1						1
Allocapnia smithi	1											1															
Allocapnia vivipara	1	1		1			1					1							1		1						1
Allocapnia zola	1					1	1					1															
Capnia vernalis					1		1																				
Paracapnia angulata	1			1	1		1												1		1						1
**Leuctridae**																											
Leuctra alexanderi	1																										
Leuctra duplicata	1																										
Leuctra ferruginea	1				1		1												1		1						1
Leuctra monticola																					1						
Leuctra rickeri	1																										
Leuctra sibleyi	1																		1		1						1
Leuctra tenella	1																										
Leuctra tenuis	1						1												1	1	1			1			1
Paraleuctra sara	1																		1		1						1
Zealeuctra claasseni	1			1	1	1	1																				
Zealeuctra fraxina	1										1																
**Nemouridae**																											
Amphinemura delosa	1				1	1	1												1	1	1			1			1
Amphinemura nigritta	1					1	1												1		1						1
Amphinemura varshava	1						1																				
Nemoura trispinosa	1						1																				
Ostrocerca albidipennis	1																				1						
Ostrocerca truncata	1					1	1												1		1						1
Prostoia completa	1																										
Prostoia similis	1				1	1	1												1		1						1
Soyedina vallicularia	1																		1	1	1			1			1
**Taeniopterygidae**																											
Strophopteryx fasciata	1				1		1																			1	
Taenionema atlanticum																											
Taeniopteryx burksi	1									1											1	1				1	
Taeniopteryx lita	1																										
Taeniopteryx maura	1			1	1		1			1									1		1						1
Taeniopteryx metequi	1																		1		1						1
Taeniopteryx nivalis	1				1																1						
Taeniopteryx parvula	1																				1						
**Peltoperlidae**																											
Peltoperla arcuata	1																				1						1

Ohio Stoneflies	Current	Need. & Claa.1925	Clark 1935	Frison 1942	Walker 1947	Ricker 1952	Gaufin 1956	Ross & Ricker 1964	Ross & Yama. 1967	Ricker & Ross 1968	Ricker & Ross 1969	Ross & Ricker 1971	Zwick 1971	Baumann 1974	1974	1976	Stark & Baum. 1978	Szc. & Stew. 1978	Tkac & Foote 1978	Robertson 1979	Tkac 1979	Full. & Stew. 1980	Szc. & Stew. 1981	Robertson 1984	Stark 1986	Beckett 1987	Fishbeck 1987
**Pteronarcyidae**																											
Pteronarcys cf. *biloba*	1																				1						
Pteronarcys dorsata	1						1																				
Pteronarcys pictetii							1																				
**Chloroperlidae**																											
Alloperla caudata	1						1												1		1						1
Alloperla chloris	1																		1	1	1			1			1
Alloperla idei	1						1																				
Alloperla imbecilla	1				1		1							1					1		1						1
Alloperla neglecta	1																				1						
Alloperla petasata	1																										
Alloperla usa	1																				1						
Haploperla brevis	1			1	1		1												1		1						1
Sweltsa mediana					1		1												1		1						1
Sweltsa hoffmani	1					1																					
Sweltsa lateralis	1																										1
Sweltsa onkos																											
**Perlidae**																											
Acroneuria abnormis	1				1		1									1				1	1			1		1	1
Acroneuria carolinensis	1																		1	1	1			1			1
Acroneuria covelli	1																										
Acroneuria evoluta	1	1	1	1	1		1																			1	
Acroneuria filicis	1			1	1		1									1					1						
Acroneuria frisoni	1	1			1		1									1					1						
Acroneuria internata	1						1																				
Acroneuria kirchneri	1																										
Acroneuria kosztarabi																											
Acroneuria lycorias	1			1	1		1									1			1	1	1			1			1
Acroneuria perplexa	1				1		1									1											
Agnetina annulipes	1																										
Agnetina capitata	1				1		1												1		1				1		1
Agnetina flavescens	1																								1		
Attaneuria ruralis	1	1					1																				
Eccoptura xanthenes	1			1	1		1														1						
Neoperla catharae	1																1										
Neoperla clymene		1			1		1														1						
Neoperla coosa	1																										
Neoperla gaufini	1																1										
Neoperla mainensis	1																1										
Neoperla occipitalis	1																1										
Neoperla robisoni	1																										
Neoperla stewarti	1																1										
Paragnetina media	1						1														1						
Perlesta adena	1																										
Perlesta cinctipes																											
Perlesta decipiens	1				1		1												1	1				1		1	1
Perlesta ephelida	1																										
Perlesta golconda																											
Perlesta lagoi	1																										
Perlesta nitida																											
Perlesta placida																					1						
Perlesta teaysia	1																										
Perlesta xube	1																										
Perlesta I–4	1						1																				

**Ohio Stoneflies**	Current	Need. & Claa.1925	Clark 1935	Frison 1942	Walker 1947	Ricker 1952	Gaufin 1956	Ross & Ricker 1964	Ross & Yama. 1967	Ricker & Ross 1968	Ricker & Ross 1969	Ross & Ricker 1971	Zwick 1971	Baumann 1974	1974	1976	Stark & Baum. 1978	Szc. & Stew. 1978	Tkac & Foote 1978	Robertson 1979	Tkac 1979	Full. & Stew. 1980	Szc. & Stew. 1981	Robertson 1984	Stark 1986	Beckett 1987	Fishbeck 1987
Perlinella drymo	1						1						1														
Perlinella ephyre	1						1						1								1						
**Perlodidae**																											
Clioperla clio	1				1	1	1												1		1		1				1
Cultus decisus	1														1					1	1			1			1
Diploperla robusta	1				1	1	1														1						1
Isoperla bilineata	1	1			1		1											1			1					1	
Isoperla burksi	1						1																				
Isoperla decepta	1						1																				
Isoperla dicala	1																				1						
Isoperla holochlora	1																										
Isoperla montana	1																		1	1				1			1
Isoperla namata																					1						
Isoperla nana	1	1			1		1														1						1
Isoperla orata	1						1																				
Isoperla richardsoni	1																										
Isoperla signata	1																				1						
Isoperla transmarina	1						1																				
Malirekus iroquois	1																										1
Total	102	7	1	11	30	10	53	1	1	2	1	13	2	1	1	5	5	1	30	10	54	1	1	10	2	6	36

Supplementary Doc1. Continued

Ohio Stoneflies	Kond. et al. 1988	Stark 1989	Young et al. 1989	Nelson 2000	Stark 2000	Stew. 2000 Grubbs & Stark	2001	Stew. & Stark 2002	Surd. 2004	Stark 2004	Stark & Baum. 2004	Kond. 2004	Grubbs 2006	Yasick et al. 2007	2008	Kond. & Kirc. 2009	Stark & Kond. 2010	Bolton 2010	2012	DeWalt et al. 2012	Stark & Kond. 2012	Grubbs et. al. 2013	Grubbs et al. 2013	Grubbs et al. 2014	Grubbs 2015	Yasick et al. 2015	Szc. & Kond. 2015
**Capniidae**																											
Allocapnia forbesi								1												1	1	1					
Allocapnia frisoni								1												1		1					
Allocapnia granulata								1												1		1					
Allocapnia illinoensis								1												1		1					
Allocapnia indianae								1												1		1					
Allocapnia mystica								1												1		1					
Allocapnia nivicola								1												1		1					
Allocapnia ohioensis								1												1		1					
Allocapnia pechumani																				1		1					
Allocapnia pygmaea								1												1		1					
Allocapnia recta								1						1						1		1				1	
Allocapnia rickeri								1												1	1	1					
Allocapnia smithi								1												1		1					
Allocapnia vivipara								1												1		1					
Allocapnia zola								1												1		1					
Capnia vernalis								1																			
Paracapnia angulata								1			1									1		1					
**Leuctridae**																											
Leuctra alexanderi																				1		1					
Leuctra duplicata																				1		1					
Leuctra ferruginea								1												1		1					
Leuctra monticola																											
Leuctra rickeri								1												1		1					
Leuctra sibleyi								1												1		1					
Leuctra tenella																				1		1			1		
Leuctra tenuis								1						1						1		1			1		
Paraleuctra sara								1												1		1					
Zealeuctra claasseni																				1		1	1				
Zealeuctra fraxina								1												1		1	1				
**Nemouridae**																											
Amphinemura delosa								1												1		1					
Amphinemura nigritta								1												1		1					
Amphinemura varshava																				1		1					
Nemoura trispinosa								1												1		1					
Ostrocerca albidipennis																				1		1					
Ostrocerca truncata			1					1												1		1					
Prostoia completa																				1		1		1			
Prostoia similis								1												1		1		1			
Soyedina vallicularia								1					1							1		1					
**Taeniopterygidae**																											
Strophopteryx fasciata						1		1												1		1					
Taenionema atlanticum								1																			
Taeniopteryx burksi						1		1												1		1					
Taeniopteryx lita																				1		1					
Taeniopteryx maura						1		1												1		1					
Taeniopteryx metequi						1		1												1		1					
Taeniopteryx nivalis																				1		1					
Taeniopteryx parvula								1												1		1					
**Peltoperlidae**																											
Peltoperla arcuata					1			1												1		1					
**Pteronarcyidae**																											
Pteronarcys cf. *biloba*																		1		1		1					
Pteronarcys dorsata				1				1												1		1					

Ohio Stoneflies	Kond. et al. 1988	Stark 1989	Young et al. 1989	Nelson 2000	Stark 2000	Stew. 2000	Grubbs & Stark 2001	Stew. & Stark 2002	Surd. 2004	Stark 2004	Stark & Baum. 2004	Kond. 2004	Grubbs 2006	Yasick et al. 2007	2008	Kond. & Kirc. 2009	Stark & Kond. 2010	Bolton 2010	2012	DeWalt et al. 2012	Stark & Kond. 2012	Grubbs et. al. 2013	Grubbs et al. 2013	Grubbs et al. 2014	Grubbs 2015	Yasick et al. 2015	Szc. & Kond. 2015
Pteronarcys pictetii				1				1																			
**Chloroperlidae**																											
Alloperla caudata								1									1			1		1					
Alloperla chloris								1	1											1		1					
Alloperla idei								1	1											1		1					
Alloperla imbecilla								1	1											1		1					
Alloperla neglecta																				1		1					
Alloperla petasata									1								1			1		1					
Alloperla usa								1	1								1			1		1					
Haploperla brevis									1											1		1					
Sweltsa mediana																											
Sweltsa hoffmani									1							1				1		1					
Sweltsa lateralis									1											1		1					
Sweltsa onkos								1																			
**Perlidae**																											
Acroneuria abnormis								1		1										1		1					
Acroneuria carolinensis								1		1										1		1					
Acroneuria covelli																				1		1					
Acroneuria evoluta								1		1										1		1					
Acroneuria filicis								1		1										1		1					
Acroneuria frisoni								1		1										1		1					
Acroneuria internata																				1		1					
Acroneuria kirchneri																				1		1					
Acroneuria kosztarabi																											
Acroneuria lycorias								1		1										1		1					
Acroneuria perplexa								1		1										1		1					
Agnetina annulipes																				1		1					
Agnetina capitata								1		1										1		1					
Agnetina flavescens								1		1										1		1					
Attaneuria ruralis										1										1		1					
Eccoptura xanthenes								1		1										1		1					
Neoperla catharae								1		1										1		1					
Neoperla clymene																				1		1					
Neoperla coosa																				1		1					
Neoperla gaufini								1		1										1		1					
Neoperla mainensis								1		1										1		1					
Neoperla occipitalis								1		1										1		1					
Neoperla robisoni																				1		1					
Neoperla stewarti								1		1										1		1					
Paragnetina media								1		1										1		1					
Perlesta adena		1						1		1										1		1					
Perlesta cinctipes								1		1																	
Perlesta decipiens		1						1		1										1		1					
Perlesta ephelida																			1	1		1					
Perlesta golconda																				1		1					
Perlesta lagoi																				1		1					
Perlesta nitida							1			1																	
Perlesta placida																											
Perlesta teaysia															1					1		1					
Perlesta xube																				1		1					
Perlesta I–4																				1		1					
Perlinella drymo	1							1		1										1		1					
Perlinella ephyre	1							1		1										1		1					

**Ohio Stoneflies**	Kond. et al. 1988	Stark 1989	Young et al. 1989	Nelson 2000	Stark 2000	Stew. 2000	Grubbs & Stark 2001	Stew. & Stark 2002	Surd. 2004	Stark 2004	Stark & Baum. 2004	Kond. 2004	Grubbs 2006	Yasick et al. 2007	Grubbs & DeWalt 2008	Kond. & Kirc. 2009	Stark & Kond. 2010	Bolton 2010	Grubbs & DeWalt 2012	DeWalt et al. 2012	Stark & Kond. 2012	Grubbs et. al. 2013	Grubbs et al. 2013	Grubbs et al. 2014	Grubbs 2015	Yasick et al. 2015	Szc. & Kond. 2015
**Perlodidae**																											
Clioperla clio								1												1		1					1
Cultus decisus								1												1		1					
Diploperla robusta								1				1								1		1					
Isoperla bilineata								1												1		1					1
Isoperla burksi								1												1		1					1
Isoperla decepta								1												1		1					1
Isoperla dicala																				1		1					1
Isoperla holochlora																				1		1					1
Isoperla montana																				1		1					1
Isoperla namata								1																			1
Isoperla nana								1												1		1					1
Isoperla orata								1																			1
Isoperla richardsoni								1																			
Isoperla signata																				1		1					1
Isoperla transmarina																				1		1					1
Malirekus iroquois												1								1		1					
Total	2	2	1	2	1	4	1	74	8	23	1	2	1	2	1	1	3	1	1	102	2	102	2	2	2	1	12
